# The Same, but Different: Shared Hallmarks and Divergent Drivers in Adult, Pediatric and Canine Glioma

**DOI:** 10.3390/curroncol33100592

**Published:** 2026-09-30

**Authors:** R. Timothy Bentley, Sagar M. Utturkar, Harish Kothandaraman, Deepika Dhawan, Danni Liu, Matthew Beyers, Min Zhang, Deborah W. Knapp, Nadia A. Lanman

**Affiliations:** 1 Department of Small Animal Clinical Science, University of Liverpool, Liverpool CH64 7TE, UK; t.bentley@liverpool.ac.uk; 2 Purdue Institute for Cancer Research, Purdue University, West Lafayette, IN 47906, USA; sutturka@purdue.edu (S.M.U.); hkothand@purdue.edu (H.K.); liu2302@purdue.edu (D.L.); minzhang@hs.uci.edu (M.Z.); knappd@purdue.edu (D.W.K.); 3 Department of Veterinary Clinical Sciences, Purdue University, West Lafayette, IN 47906, USA; ddhawan@purdue.edu; 4 Department of Statistics, Purdue University, West Lafayette, IN 47906, USA; 5 Department of Epidemiology and Biostatistics, UC Irvine, Irvine, CA 92697, USA; 6 Cancer Data Science Initiatives, Leidos Biomedical Research, Inc., Frederick National Laboratory for Cancer Research, Frederick, MD 21701, USA; matthew.beyers@nih.gov; 7 Department of Comparative Pathobiology, Purdue University, West Lafayette, IN 47906, USA

**Keywords:** genomics, transcriptomics, glioblastoma, methylation, molecular, mutation, signaling, tumor microenvironment, comparative oncology

## Abstract

Malignant gliomas are the most common primary brain tumors, with dismal outcomes. Pet dogs have been proposed as a valuable model for studying gliomas. Here, we compare spontaneously occurring gliomas from adult and pediatric human and canine patients, focusing on the tumor microenvironment (TME) and the canonical pathways of human glioma dysregulation (RTK/RAS/PI(3)K, p53 and Rb). Transcriptomic, genomic, and methylation data from adult, pediatric, and canine samples were evaluated. Across cohorts, aberrant signaling in the three canonical pathways correlated with increasing tumor grade. Canine high-grade glioma (HGG) showed extensive pathway dysregulation, especially when incorporating differentially expressed genes such as TP53. PDGFRA was a key driver of glioma in purebred brachycephalic (“short-skulled”) dogs. IDH1 mutations and hypermethylation were concentrated in adult tumors. Both species exhibited increased neutrophils, M2 macrophages, and regulatory T cells, coupled with depletion of key antitumor effectors. PD-1 and PD-L1 immune checkpoint biology was similarly preserved. Canine glioma seems to occupy a “middle ground” between pediatric and adult. Methylation patterns mirrored pediatric glioma, yet immune infiltration and pathway activation resembled adult HGG. Complex heterogeneity between patients was a major similarity between species. Despite different driver mutations, dogs and humans converge on a similar malignant, immune-suppressed TME.

## Simple Summary

Gliomas are a type of brain tumor affecting both children and adults, with outcomes that have remained similar over many decades. One major barrier to the development of novel therapies is a lack of animal models that reliably predict which treatments will work in human patients. Pet dogs naturally develop gliomas, but it was previously unclear how closely canine and human gliomas resemble each other at the molecular and cellular levels. This study identifies many similarities in terms of genetic changes, gene activity, and immune features across adult (human), pediatric (human), and canine gliomas. Despite certain differences in the specific mutations involved, dogs and humans showed strikingly similar disruptions to key gene pathways that lead to cancer, and similar immune environments within tumors. These findings support using pet dogs with naturally occurring gliomas to test new glioma treatments, potentially accelerating the development of more effective treatments for both species.

## 1. Introduction

Gliomas are a heterogeneous group of primary brain tumors spanning a wide range of malignancy. At the most aggressive end of the spectrum, glioblastoma (GBM) carries a median survival rivaling the deadliest of human cancers and remains almost invariably recurrent and fatal. In children, brain tumors have the shortest survival of any pediatric cancer, and gliomas have recently surpassed leukemia to cause more deaths than any other pediatric cancer.

The outcome of adult and pediatric glioma has remained similar for decades, partly due to the challenge of finding a pre-clinical model that accurately predicts the efficacy of novel therapies. Frequently, novel glioma therapies appear successful in cell line or immunodeficient mouse xenograft studies but are subsequently found to be ineffective in human clinical trials [1]. When comparing results of preclinical murine studies and human clinical trials for 11 novel therapies for recurrent GBM, there was no significant association between murine survival and human survival in phase II studies [2]. Even worse, improved murine tumor shrinkage correlated with shorter human survival, although this was also non-significant. These data highlight that high-grade gliomas continue to carry a poor prognosis, in part because the current developmental pipeline for new therapies does not predict which therapies will be effective when translated into human patients.

“Companion dogs” (privately owned dogs) with spontaneously arising gliomas are an established model for human glioma, including GBM [3,4,5,6,7,8,9,10,11,12,13,14,15,16,17,18,19,20,21,22]. Canine gliomas have attracted support from the NIH and other leading biomedical agencies in the development of immunotherapies [23,24,25,26,27,28,29,30,31,32,33] and other novel therapies for translation into human patients [34,35,36,37,38,39]. Spontaneously occurring glioma in dogs recapitulates key biological features of human glioma that are often missing, even with in vivo laboratory models. Canine gliomas display substantial inter-patient variation in tumor type, grade, location, patient age, sex, and genetic background. Both species display predispositions yet variations, e.g., high tumor grade and middle-aged onset. Critically, canine and human gliomas exhibit marked intra-tumoral heterogeneity, with regions of dense neoplastic cells, angiogenesis involving endothelial proliferation, necrosis, and invasion into adjacent brain tissue. These are features that in vitro models cannot faithfully reproduce [22,40]. Rodent models typically use genetically identical tumors implanted into genetically identical hosts. A lack of inter-patient and intra-tumoral variation is a key limitation when modeling GBM, where heterogeneity is a key barrier to developing universally effective therapies.

Crucially for immunotherapy development, gliomas in human and canine patients spontaneously develop in immunocompetent hosts with unmodified immune systems “educated” by antigens present in human households [41,42]. An established glioma represents a failure of host immunological defense mechanisms, facilitated by tumor-induced immunosuppression [1]. Immunodeficient mice implanted with human-patient-derived xenografts lack the full range of interactions between pro- and anti-inflammatory cells in the peritumoral milieu. In-depth understanding of the naturally occurring glioma tumor microenvironment (TME) is essential [33].

When translating across species, it is important to recognize differences in disease classification. Regarding glioma diagnosis, veterinary and human criteria are broadly aligned [6,43,44,45]. However, differences exist in the features used to assign a grade and to diagnose GBM specifically. The human cohorts used in our study comprise glioma cases diagnosed and graded according to earlier WHO CNS tumor guidelines. The WHO classification underwent serial revisions in 2007 (4th ed.), 2016 (4th ed. revised), and 2021 (5th ed.) [44,46,47]. Importantly, rather than reclassifying our human cohorts under the current (5th ed.) WHO criteria, we have intentionally retained original diagnoses. This approach preserves the clinical and pathological context in which these tumors were characterized and avoids the circularity of applying molecular criteria retrospectively to cohorts for which a complete contemporary workup is unavailable. We do, however, directly address the two most consequential classification differences. First, the current WHO system designates IDH-wildtype (IDHwt) status as a defining criterion for GBM (grade 4) [44], whereas our human dataset predates this requirement and therefore includes some IDH-mutant (IDHmut) grade 4 tumors; we explicitly account for this in our molecular analyses. Second, current WHO guidance uses immunohistochemistry (IHC) to determine IDH mutation status, whereas we directly use sequencing of IDH1 and IDH2, a more sensitive approach that is fully concordant with IHC in the vast majority of cases.

The only WHO veterinary brain tumor classification dates to 1999 [43]. Fortunately, there has been much subsequent characterization of canine glioma pathologically and immunohistochemically [6,45,48,49,50,51,52,53,54]. Most notably, the Comparative Brain Tumor Consortium (CBTC) of the NIH convened a glioma pathology board, comprising veterinarians and physician neuropathologists. The board produced a system for classifying (astrocytoma, oligodendroglioma, undefined) and grading (high, low) canine glioma [6]. This system has been widely adopted in translational canine–human research and was applied throughout the present study.

In canine–human translational brain cancer research, dogs are divided into two populations: short-nosed (brachycephalic) breeds with a strong predisposition to glioma and longer-nosed (mesocephalic or dolichocephalic) breeds, referred to as “non-brachycephalic” throughout this study. Brachycephalic breeds have been bred to have human-like skull morphology. The majority of gliomas occur in a single clade of closely related brachycephalic breeds (e.g., French bulldogs, Boston terriers, boxer dogs) [6,15]. In total, 53% of canine gliomas occurred in just five brachycephalic breeds [55], and French bulldogs had a glioma odds ratio of 61 [56]. Brachycephalic dogs are prone to the narrowing of upper airways (brachycephalic obstructive airway syndrome); the associated hypoxia is of potential relevance in brain tumor biology [57].

The purpose of this study was to document similarities and differences between genetic, molecular, and immunological signatures in human and canine glioma, including brachycephalic and non-brachycephalic sub-groups, to support future translational canine–human research. We find that although canine and human tumors differ in initiating mutations, convergent evolution driven by the brain microenvironment and an intact immune system results in a shared malignant phenotype and immunosuppressive TME that cannot be readily recapitulated in other cross-species models.

## 2. Materials and Methods

### 2.1. Study Overview

Spontaneously occurring gliomas from adult human, pediatric human, and adult canine patients were utilized (Table 1). Adult and canine brain tissue data, derived from cancer-free individuals, served as respective controls. The glioma subjects and controls in each individual analysis are specified in Table S1, and the associated metadata are included in Tables S2–S5.

### 2.2. Classification of Glioma Tumors

For all three cohorts (adult, pediatric, canine), grade 1 and 2 gliomas were classified as low-grade glioma (LGG), whereas grade 3 and 4 gliomas (including GBM) were classified as high-grade glioma (HGG). Only astrocytoma, glioblastoma, oligodendroglioma, and oligoastrocytoma were included. Astrocytomas and glioblastomas were analyzed together as astrocytomas. In dogs, oligoastrocytomas have been retitled “undefined glioma” [6]. Accordingly, across all three cohorts, each of oligoastrocytoma, mixed glioma, and undefined glioma was included in the oligoastrocytoma group. Ependymoma and all other tumor types were excluded.

Within each cohort (adult, pediatric, and canine), the following analyses were performed on LGG and HGG separately.

### 2.3. Data Collection

#### 2.3.1. Tumor Data

Adult pre-processed mutations, copy-number variations, methylation, and transcriptomic data were obtained from the NCI Genomic Data Commons (GDC) under the TCGA-GBM and TCGA-LGG studies, both generated by the TCGA Research Network via the R package TCGAbiolinks (v2.27.2) [58] and recount3 (v1.4.0) [59]. Methylation beta-values from the adult samples were obtained using the Shiny Methylation Analyses Resource Tool (SMART, accessed on 21 September 2023) [60]. Pediatric whole-genome, somatic mutation, and transcriptomic gene-expression (RSEM) data were obtained from the Kids First Data Resource Center (Kids First) of the Children’s Brain Tumor Network (CBTN) study, accessed via the CAVATICA platform at the Cancer Genomics Cloud (CGC) by Seven Bridges Genomics. Canine whole-genome, whole-exome, methylation, and transcriptomic datasets were obtained from the NCI-Integrated Canine Data Commons (ICDC) under study accession GLIOMA01. The canine patients were adult companion dogs (ages 1.2 to 17 years, mean 8.5 years) presenting to North American university veterinary hospitals for the diagnosis and management of spontaneously occurring gliomas.

The canine gliomas in this study were previously analyzed by Amin et al. [3]. The complete genomic data from these canine cases are available through the ICDC website (https://caninecommons.cancer.gov/). Compared with Amin et al.’s research, this study used the same adult glioma database (TCGA), with additional cases added in recent years, and a different, larger cohort of pediatric cases (KidsFirst). Cancer-free cohorts were used as “true-normal” controls, rather than contralateral brain tissue for canine glioma cases.

#### 2.3.2. Normal Data

Transcriptomic data used in this analysis were obtained from healthy individuals (i.e., true-normal samples) and excluded adjacent-normal samples from glioma patients. Somatic mutations, copy-number variations, and methylation calling were performed using matched normal data from respective subjects. Adult true-normal cerebral data were retrieved from the Genotype-Tissue Expression (GTEx) Portal [61]. Pediatric normal brain data were not available. Canine true-normal brain tissue data were gathered from 12 dogs of varying breeds and ages, including publicly available data from BarkBase (*n* = 5) [19]. The BarkBase data were normal frontal cortex data from a Yorkshire terrier, a Labrador-mix, a Newfoundland, and two Malinois, aged 2 to 16 years. Additionally, normal flash-frozen cerebral tissue was sequenced at Purdue University, harvested from 5 normal adult laboratory beagles (donated by Dr. S. Platt, University of Georgia) and 2 Purdue University Veterinary Hospital patients (Australian Cattle Dog and mix-breed, aged 5 and 8 years, euthanized for disease unrelated to glioma, with necropsy revealing acute spinal cord injury, and bladder cancer without brain metastasis, respectively).

### 2.4. Data Availability

The RNA sequencing data for canine normal brain tissue samples generated in this study have been deposited in the Gene Expression Omnibus (GEO) under accession number GSE330017.

### 2.5. Data Processing

All analyses were performed on the CGC and CAVATICA platforms unless otherwise stated. Germline variations from 722 canid genomes (NCBI Bio project: PRJNA448733) from the Dog10K project [62] were employed as a canine germline resource wherever applicable.

#### 2.5.1. Somatic Variation Processing

Canine whole-genome and exome-aligned BAMs from the same patients were combined using BAMTools merge (v2.4.0) [63]. Somatic Single Nucleotide Polymorphisms (SNPs) and insertion–deletion events (Indels) were identified using VarScan2 (v2.3.9) [64] using matched normals wherever applicable. SNPs and indels from VarScan2 were combined using the smart_variants pipeline on the CGC platform. VarDict (v1.5.1) [65] was used to identify somatic SNPs and Indels for canine samples without matched normals. Variants were annotated with the variant effect predictor (VEP) [66] using the CanFam3.1 genome. Annotated VCFs were converted to MAF files using the vcf2maf tool. Putative germline mutations were filtered out using the DOG10K germline resource [62] and the bcftools isec complement function (v1.8). Pediatric and adult SNPs and Indels were directly available in the mutation annotation format (MAF). Human and dog IDH1 protein sequences (UniProt [O75874, 0A2Z5XAE6]) were aligned with Kalign v3.3.1 [67] via the EMBL-EBI Job Dispatcher [68] using default parameters (gap open = 11.0, gap extension = 4.0, terminal gap extension = 2.0), and inspected for conservation at the R132 hotspot.

Copy number variations (CNV) were identified using CNVKit (v0.9.3) [69]. The CNVkit reference was prepared by pooling matched normal samples (by maximum sequencing depth and excluding FFPE whenever possible). The canine CNVkit reference included 22 matched normals, and the pediatric reference included 29 matched normals. Tumor samples were screened against their respective references using the CNVKit copy-number calling pipeline. Adult CNV calls were directly available at TCGA as processed data. For canine and pediatric data, CNVs with absolute copy number loss (CN = 0) were denoted as homozygous deletion and (CN ≥ 5) as high amplification and selected for subsequent analyses. Adult CNVs marked as Gain (roughly matches absolute copy number ≥ 5) or Loss (roughly matches absolute copy number = 0) were selected for subsequent analyses.

#### 2.5.2. Germline Variation Processing

Canine germline variations were identified using matched normal data from each patient by the GATK HaplotypeCaller [70,71,72] mode (v4.1.1.0) and followed by variant filtering via the variant quality score recalibration (VQSR) pipeline implemented in GATK (v4.6.0.0) and a panel of 722 dogs (Dog10K) [62]. Variations marked as “PASS” were selected and annotated with CanFam3.1 using VEP. Only the variations with impact categorized as “HIGH” (entirely disruptive, protein truncation, loss of function, etc.) and “MODERATE” (changes protein effectiveness, is disruptive) by VEP were used for subsequent analysis. Mutations in PRPS2 were mapped to protein domains by comparing amino acid positions to annotated PRPP synthetase domains using domain annotations from UniProt and InterPro. Variant locations were evaluated relative to the conserved PRPP synthetase catalytic core and C-terminal regulatory regions defined for PRPS family proteins.

#### 2.5.3. Variation Analysis

Analysis of processed data (MAF, CNV) was performed on Purdue community computing clusters using R (version 4.3.3) and the maftools package (v2.18.0) [73]. MAF files for somatic variations, along with respective CNV and clinical data, were imported into maftools, and individual cohorts (adult, pediatric, canine) were created. MAF files for canine germline variations were imported separately, and a germline cohort was created. Only non-synonymous variants were considered for downstream analysis. Required sub-cohorts were created using the subset function in maftools. Differentially mutated genes (DMGs) between the two cohorts were determined using maftools. Maftools functions, such as createOncoMatrix for mutation statistics and oncoplot for plotting mutations, were employed. Lollipop plots showing specific amino-acid substitutions within protein domains were created using the R package ggplot2.

#### 2.5.4. Methylation Analysis

Canine methylation BAMs were processed with Bismark [74] to generate coverage information on methylation sites. Differential methylation calling was performed by contrasting individual canine samples with the 3 methylation-normal samples, identifying sites and genomic windows using MethylKit (v1.16.1) [75]. Hyper- and hypo-methylated regions were annotated with R package Annotatr (v.1.16.0) [76].

#### 2.5.5. RNA-Seq Analysis

RNA was extracted from normal canine brain samples collected from dogs undergoing euthanasia with no brain disease using the AllPrep DNA/RNA kit following the manufacturer’s instructions (Qiagen, Germantown, MD, USA). Samples collected were histopathologically confirmed to be normal brain tissue. RNA-seq was performed using strand-specific libraries, and raw data were aligned to the CanFam3.1 reference using the STAR aligner (v2.7.10b) [77]. Tumor transcriptomic BAMs were available through ICDC. A raw counts matrix for all canine samples was generated by quantification via HT-Seq (v0.6.1) [78]. Transcript per million (TPM) counts were determined using the R package DGEobj.utils (v1.0.6) with gene lengths from bioMart for the respective species. For duplicate gene symbols, the entry with the highest row sum of TPM values was selected for subsequent analysis. Differentially expressed genes (DEGs) were identified using the edgeR package (v3.36.0) [79] by conducting gene-wise tests under the quasi-likelihood GLM framework. *p*-values were corrected using the Benjamini and Hochberg method [80], and genes with an adjusted *p*-value < 0.05 were considered significant. Gene Ontology (GO) and pathway enrichment were assessed using Qiagen’s Ingenuity Pathway Analysis (IPA) and the R package clusterProfiler (v4.10.1) [81].

Cell-type proportions were estimated using Cibersort (v1.04) [82] and quanTIseq (v1.10.0) [83], and tumoral, immune, and stromal scores were estimated using the ESTIMATE algorithm (v1.0.13) [84] with the immune cell deconvolution R package immunedeconv (v 2.1.0) [85] and TPM counts. Single-sample GSEA (v2.0) (ssGSEA) [86,87] was run with RNA-seq TPM counts and custom gene panels developed using canine and human orthologs. Custom gene sets used for enrichment analysis were obtained from KEGG [88] and the canine IO panel gene sets [89], accessed via Brucker, while immune-suppressing and immune-enhancing gene sets were manually curated [90]. A positive or negative ssGSEA score indicates that the gene set is coordinately upregulated or downregulated, respectively. ssGSEA scores are relative and can be used to compare gene set activity across samples to identify trends within the cohort. Heatmaps and custom figures were created using the R packages ggplot2 (v3.5.1) [91], ggpubr (v 0.6.0) [92], and ComplexHeatmap (v 2.18.0) [93].

Cross-species data harmonization was performed using orthologous genes derived from the Ensembl BioMart database. Canine genes were mapped to corresponding human orthologs, and only ortholog-mapped genes were retained for downstream immune deconvolution analysis. Because Cibersort, quanTIseq, and ESTIMATE rely on human-derived signatures, all analyses of canine samples were conducted using human-ortholog-transformed expression matrices. The suitability of this approach was assessed by evaluating the TIL10 signature. Batch corrections were not performed because batch metadata was not consistently available across the public datasets used.

#### 2.5.6. Key Definitions

Definitions utilized in this study include abnormal signaling (mutation or CNV), gene expression (as assessed by RNA-seq), and the 3 canonical oncogenic pathways of human GBM (the RTK/RAS/PI3K, p53, and Rb1 pathways) [94].

Key genes were selected from the 3 canonical pathways, the WHO classification of human glioma [44], the immune microenvironment, and the known glioma prognostic indicators. CNV ≥ 5 was considered high amplification, whereas CNV = 0 was considered a homozygous deletion. For methylation, a beta value < 0.30 was considered hypomethylation, and a beta value > 0.50 was considered hypermethylation.

## 3. Results

### 3.1. Case Information

A total of 1035 adult, 258 pediatric, and 81 canine gliomas met the inclusion criteria (Table 1 and Tables S2–S5). Data availability (WGS, CNV, RNA-seq, methylation) for each cohort is denoted in Table S1.

All adult cases reportedly underwent glioma sampling during surgical biopsy [94], which is nearly always performed after the commencement of glucocorticoids. Pediatric glioma tissue was reportedly collected post-mortem [94], which typically follows therapeutic interventions (e.g., radiation therapy) and protracted symptomatic therapy (e.g., glucocorticoids).

All canine cases underwent glioma sampling at necropsy. Survival data were available for 67 dogs: 43 who received no definitive therapy and 24 who did. It is likely that some received symptomatic glucocorticoids prior to euthanasia; 25 of them were euthanized on the day of the diagnostic MRI, and four were euthanized the following day. The interval between any glucocorticoids and euthanasia was therefore very brief. The other 14 dogs in this group likely received symptomatic glucocorticoids and survived for 3–349 days. The group of 24 dogs who received definitive therapy (surgery, radiation therapy, experimental therapies, or combinations thereof) survived for 2–831 days. Survival for these definitive therapy patients (median, 125 days) was significantly longer than for all other dogs (median, 1 day) (*p* < 0.0001). For the 14 dogs without survival data, one underwent surgery, one was euthanized without therapy, and 12 had no treatment data.

### 3.2. All 3 Canonical GBM Pathways Are Affected by Abnormal Signaling and Differential Gene Expression in Human and Canine Glioma

**Figure 1:**
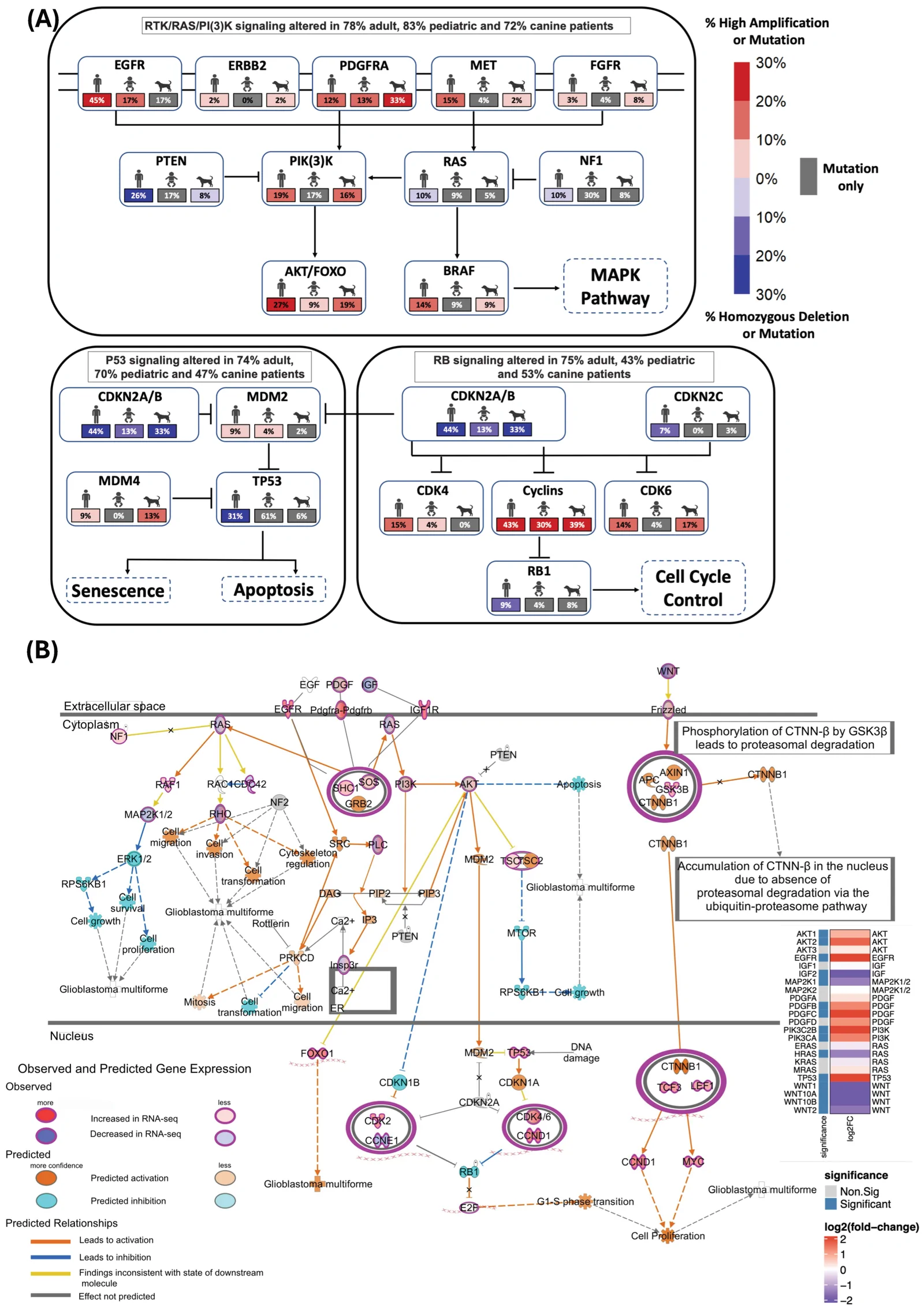
The three canonical oncogenic pathways of GBM: abnormal signaling and differentially expressed genes. (**A**) Abnormal signaling (mutations, high amplification, or homozygous deletion) in adult, pediatric, and canine high-grade glioma (HGG) (whole genome and whole exome). Three signaling pathways are shown: RTK/RAS/PI(3)K, p53, and RB signaling. Total % alteration is at the top of each pathway. For each gene, three boxes denote the % alteration (mutation, high amplification, or homozygous deletion) across adult, pediatric, and canine cohorts, respectively. Red denotes high amplification and mutation, blue denotes homozygous deletion and mutation, and darker shades correspond to a higher % alteration. A gray color indicates that neither amplification nor deletion was detected for that gene. There was marked amplification spanning the RTK pathway, of the TP53 inhibitors MDM2 and MDM4, and of the cyclins in the RB pathway. There was marked deletion of tumor suppressors CDKN2A/B. CDK4 was the only gene across all three pathways that was unaffected by mutation or CNV in canine HGG. (Table S6 lists the 35 cyclins represented in this figure). An “X” placed across a relationship line (edge) indicates a non-occurring or blocked interaction in disease. (**B**) Differentially expressed genes (RNA-seq) in canine HGG. All three pathways showed statistically significant abnormal gene expression in canine HGG compared with the normal canine brain. An outer purple circle denotes molecules that are significantly differentially expressed (*p* ≤ 0.05). This includes individual molecules (e.g., PDGF, IGF) and complexes of genes (e.g., CCND1 binding to CDK4/8). The stronger the internal red shading, the stronger the upregulation (e.g., EGFR, PDGFRs alpha and beta, TP53); versus the stronger the internal blue shading, the stronger the downregulation (e.g., WNT, RAS, IGF). Internal shading (of red versus blue) is only applied if the differential expression exceeds logFC ≥ 2.0. Predicted activation (MDM2, Mitosis) and inhibition (Rb1, mTOR, Apoptosis) are shown in brown and cyan, respectively. The heatmap denotes a clean expression pattern (red: upregulated, blue: downregulated) for individual genes (names on left) and molecules (names on right) mapped by IPA. Statistical significance (FDR < 0.05) for each gene is denoted by blue/gray row annotation on the left of the heatmap. * denotes multiple duplicates or multiple instances/family members of that molecule present or represented in the pathway network.

Our results allow parallel comparison of molecular aberrations across the three canonical pathways of GBM (RTK/RAS/PI3K, p53, and Rb1 pathways [94]) in adult, pediatric, and canine gliomas (Figure 1, Table 2, Table 3 and Table S6). Across human and canine cohorts, the frequency and intensity of canonical pathway disruption increased with tumor grade (Table 2), manifesting as abnormal signaling (mutations and CNVs, Figure 1A and Figure S1). Canine HGG showed marked differential gene expression across all three pathways (RNA-seq, Figure 1B).

In HGG, abnormal signaling was highly prevalent in the RTK/RAS/PI3K pathway, affecting 72–83% of adult, pediatric, and canine cases (Figure 1A). Cross-species similarities included loss of *PTEN* and *NF1* in each of the adult (mutation and deletion), pediatric (high mutation), and canine (mutation) contexts. Canine HGG showed increased expression of *PDGF* and its receptors, PDGFR-alpha (*PDGFRA*) and PDGFR-beta (Figure 1B). Upstream of *EGF*, *ID-1* overexpression was strongest in canine glioma (Table 3). The *TP53* mutation rate was a notable species difference: 31% in adults and 61% in pediatric cases versus 6% in canine cases (Figure 1A). Nonetheless, TP53 expression was elevated in both adult and canine glioma (Table 3). There was high amplification of p53 inhibitors (particularly *MDM2* in adult glioma and *MDM4* in canine glioma), intensified by *CDKN2A/B* deletion (Figure 1A). Rb1 pathway molecular aberrations were dominated by those affecting the cyclins (all 3 HGG cohorts) or *CDKN2A* (especially adults) (Figure 1A). Table S6 lists the gene symbols and molecules (including cyclins) used in Figure 1A, and Table S7 lists those used in Figure 1B.

**Table 3:** Copy number variation (CNV, number of samples with homozygous deletion (0 copies) or high amplification (≥5 copies), mutation rate, expression (RNA-seq), and methylation in adult, pediatric, and canine glioma (LGG and HGG). No normal tissue data were available for pediatrics.

*The 3 Canonical Pathways*		Adult	Pediatric	Canine
PDGFRA	CNV rate Mutation rate RNA-seq (fold-change)	10% (88/887)	2% (2/110)	15% (12/79)
		3% (23/880)	4% (5/118)	22% (17/79)
		+2.81 *	–	+5.85 *
EGFR	CNV rate Mutation rate RNA-seq (fold-change)	36% (316/887)	3% (3/110)	0% (0/79)
		13% (117/880)	3% (3/118)	15% (12/79)
		+4.50 *	–	+3.99 *
Rb1	CNV rate Mutation rate RNA-seq (fold-change)	5% (45/887)	0% (0/110)	0% (0/79)
		5% (42/880)	1% (1/118)	6% (5/79)
		+1.70 *	–	−0.68 *
TP53	Mutation rate RNA-seq (fold-change)	41% (358/880)	14% (16/118)	5% (4/79)
		+2.72 *	–	+2.40 *
***WHO glioma classification***				
IDH1	CNV rate Mutation rate RNA-seq (fold-change)	2% (21/887)	0% (0/110)	0% (0/79)
		47% (417/880)	2% (2/118)	4% (3/79)
		+2.20 *	–	+0.50
ATRX	CNV rate Mutation rate RNA-seq (fold-change)	26% (230/887)	1% (1/110)	0% (0/79)
		25% (222/880)	8% (9/118)	4% (3/79)
		+0.37 *	–	+1.64 *
***The Immune TME***				
PD-1	RNA-seq (fold-change) Methylation (beta value)	+1.99 *	–	+0.50
		0.6537 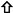	–	0.2443 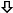
PD-L1	CNV rate Mutation rate RNA-seq (fold-change) Methylation (beta value)	0% (0/887)	–	1% (1/79)
		0% (0/880)	–	1% (1/79)
		+0.75 *	–	+1.06 *
		0.0573 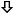	–	0.0109 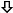
HLA-G	RNA-seq (fold-change)	+1.11 *	–	–
CXCL2	RNA-seq (fold-change)	−0.17 *	–	−1.22 *
***Key prognostic markers***				
MGMT	CNV rate RNA-seq (fold-change) Methylation (beta value)	5% (41/887)	0% (0/110)	0% (0/79)
		−0.18 *	–	+1.45 *
		0.8957 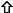	–	0.1899 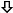
CXCL14	CNV rate Mutation rate RNA-seq (fold-change)	3% (25/887)	0% (0/110)	0% (0/79)
		0% (0/887)	0% (0/118)	0% (0/79)
		−1.03 *	–	−2.32 *
ID-1	CNV rate Mutation rate RNA-seq (fold-change)	5% (43/887)	0% (0/110)	0% (0/79)
		0% (0/880)	0% (0/118)	0% (0/79)
		+1.28 *	–	+1.85 *

**Figure 2:**
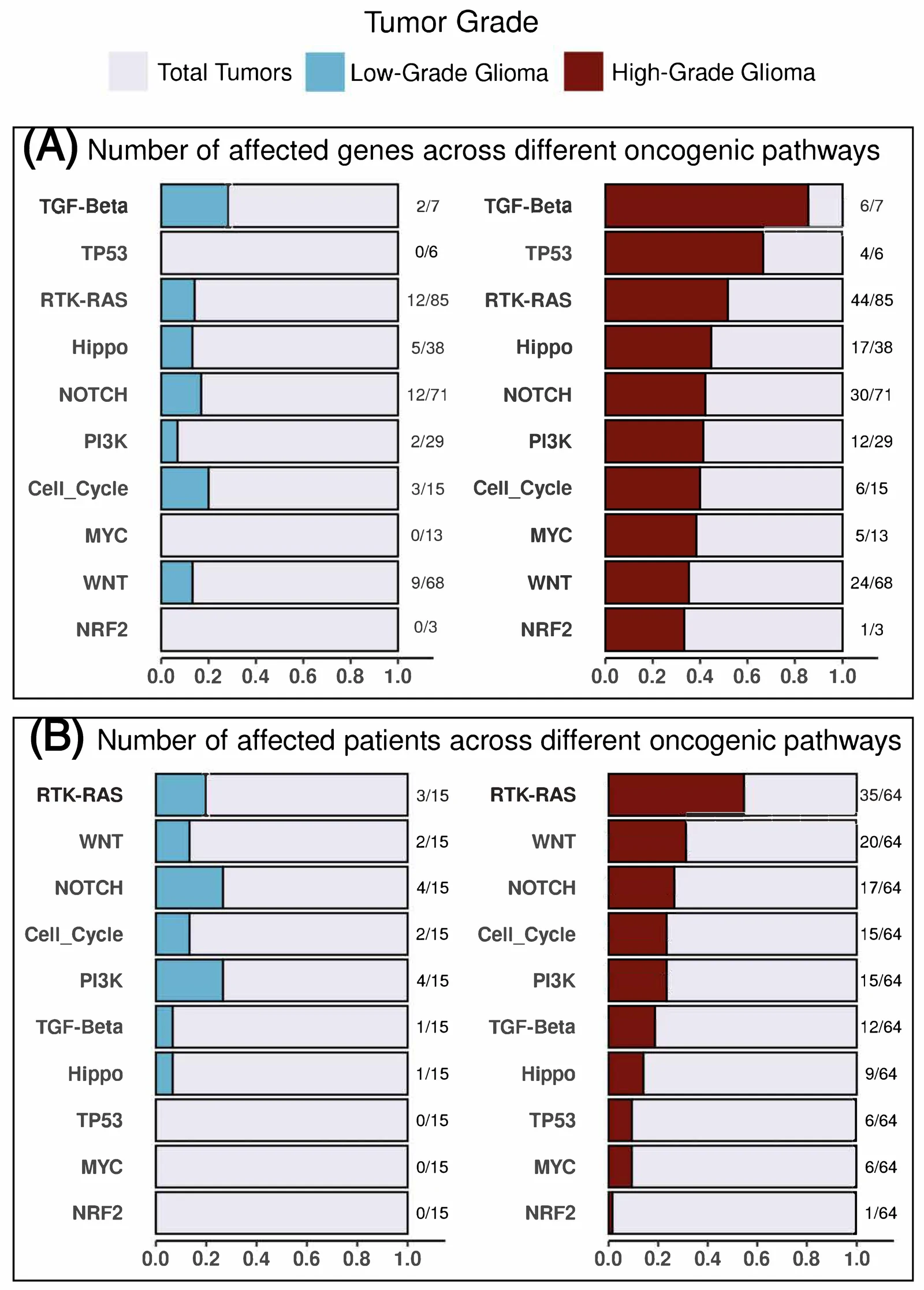
Mutational burden in canine glioma. (**A**) The number of mutated genes in each oncogenic signaling pathway. (**B**) The number of cases incorporating a mutation within each oncogenic signaling pathway. A total of 64 high-grade and 15 low-grade canine gliomas are presented. In all, six of the seven genes in the TGF-beta pathway were mutated in at least one dog with high-grade glioma, and these changes were concentrated in just 12 cases. A total of 44 of the 85 genes in the RTK-RAS sub-pathway were affected in high-grade glioma across 35 cases. The proportion of each pathway and the fraction of cases affected (x-axis) were generally higher in HGG than in LGG. However, the PI3K pathway (downstream of RTK-RAS) carried at least one mutation in 4 of the 15 (27%) low-grade cases.

* denotes significance (FDR < 0.05) compared to normal brain tissue; down arrows denote hypomethylation (beta value < 0.30); and up arrows denote hypermethylation (beta value > 0.50). Other oncogenic pathways widely affected in canine glioma are shown in Figure 2. Over half of the genes in each of the TGF-beta, p53, and RTK-RAS pathways were mutated in ≥1 HGG (Figure 2A). Similarly, the proportion of canine tumors carrying at least one mutation in each oncogenic pathway was much higher in HGG, except for *PI3K* (Figure 2B). CNV occurred in both low- and high-grade canine gliomas (Figure S1).

**Figure 3:**
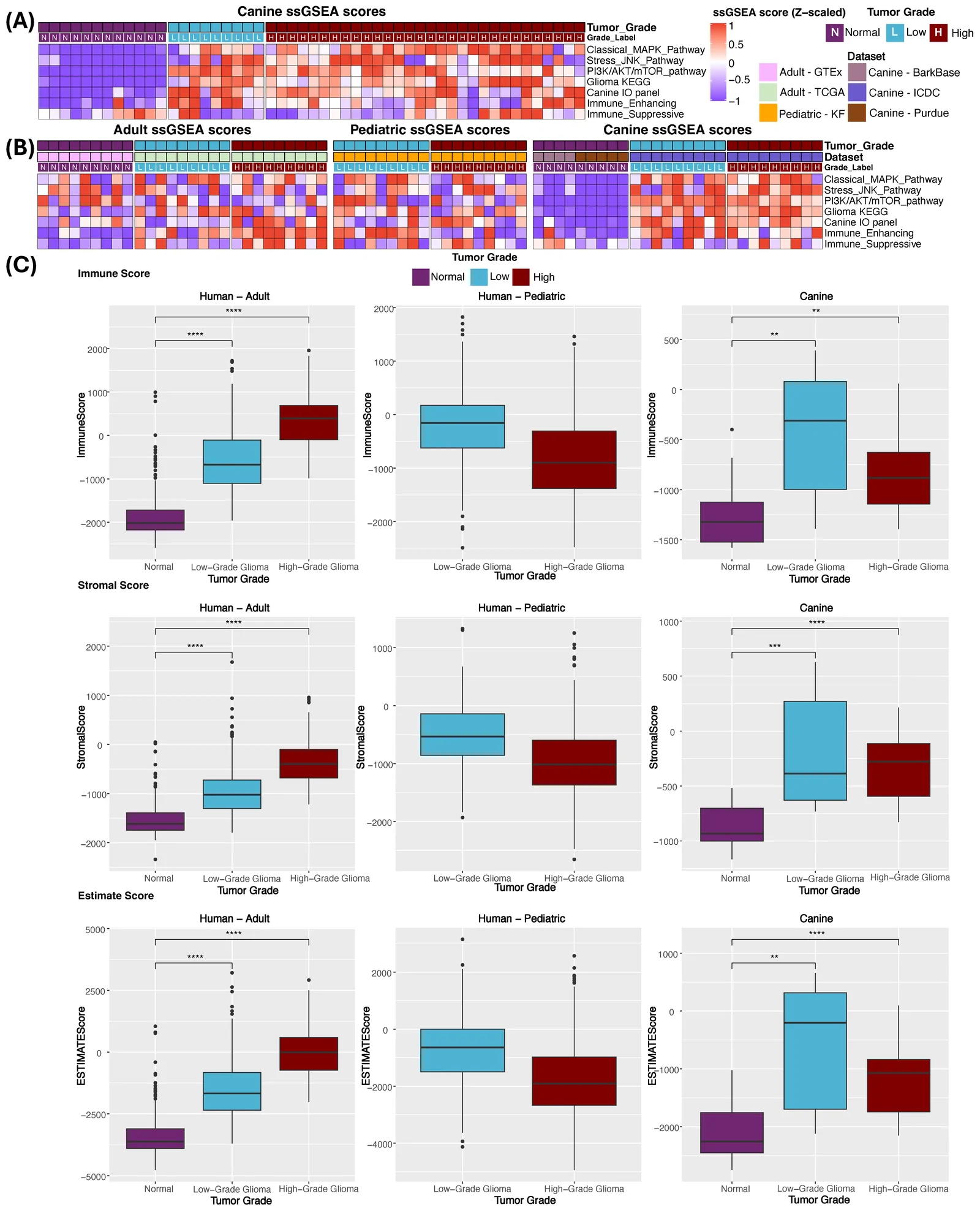
Adult, pediatric, and canine glioma ssGSEA pathway, immune, and stromal scores. (**A**) Z-scaled ssGSEA scores were compared across tumor and normal samples within individual species and individual gene sets. Nine samples were randomly selected from each available group for this visualization to enhance readability. (**B**) ssGSEA scores are represented across the entire available canine RNA-seq dataset (Normal n = 12, LGG n = 9, and HGG n = 30). Negative scores (blue) denote weaker enrichment, while positive scores (red) denote stronger enrichment of the given pathway across samples within each species. Disease grade and dataset details are indicated as annotations at the top of the heatmap. Enrichment of the JNK and PI3K pathways is observed when comparing normal vs. low-grade glioma vs. high-grade glioma across species. The difference between normal and tumor in MAPK is most pronounced for canine HGG. The PI3K/AKT/mTOR pathways are noteworthy, as they overlap with the RTK/RAS/PI3K pathway, and downstream action through the HIF1alpha and the p53 pathways stimulates angiogenesis and cell survival, respectively. Gene sets associated with gliomas (KEGG and IO) are also enriched across species. Immune-suppressive genes are enriched in high-grade canine and adult gliomas. In contrast, immune-enhancing genes are more enriched in low-grade pediatric and canine gliomas and in high-grade adult glioma. (**C**) Immune, stromal and estimate scores in adult, pediatric and canine glioma. Comparison of mean immune, stromal and estimate scores between normal, high-grade glioma and low-grade glioma within each cohort. An unpaired non-parametric Wilcoxon rank-sum test was performed to compare the means; significance (Benjamini–Hochberg adjustment) of mean differences is denoted by asterisks (** *p* < 0.01, *** *p* < 0.001, **** *p* < 0.0001).

Single-sample gene set enrichment analysis (ssGSEA; Figure 3) demonstrated cross-species enrichment of MAPK, JNK, and PI3K/AKT/mTOR signaling, which increased with grade across all three cohorts. The activation of the MAPK pathway, in comparison to the normal brain, was strongest in canine HGG (Figure 3B). Gene sets characterized as important in human glioma (KEGG hsa05214, referred to as “Glioma KEGG” in the figures [88]) were enriched in canine gliomas, with even higher enrichment within HGG.

### 3.3. IDH Mutation and Hypermethylation Are Concentrated in Adult Glioma

IDH1 was both heavily mutated and overexpressed in adult glioma (Table 3, Table S8). The adult human *IDH1* mutations were mainly R132H (90.7%); the remaining mutation events were R132G, R132C, or R132S. The three canine *IDH1* mutation events were all R132C, with no change in expression. IDH1^R132C^ in canines, like IDH1^R132H^ in human gliomas, disrupts the conserved active-site arginine required for substrate coordination (confirmed by multiple sequence alignment, Figure S2), resulting in comparable neomorphic 2-hydroxyglutarate-producing activity [95]. They were more common in LGG (2/15, 13.3%) than HGG (1/64, 1.6%). *PD-1*, and *MGMT* were hypermethylated in adults and hypomethylated in dogs, although *PD-L1* was hypomethylated within both species (Table 3).

### 3.4. PDGFRA Somatic Mutations Are Concentrated in Brachycephalic Dog Glioma

**Figure 4:**
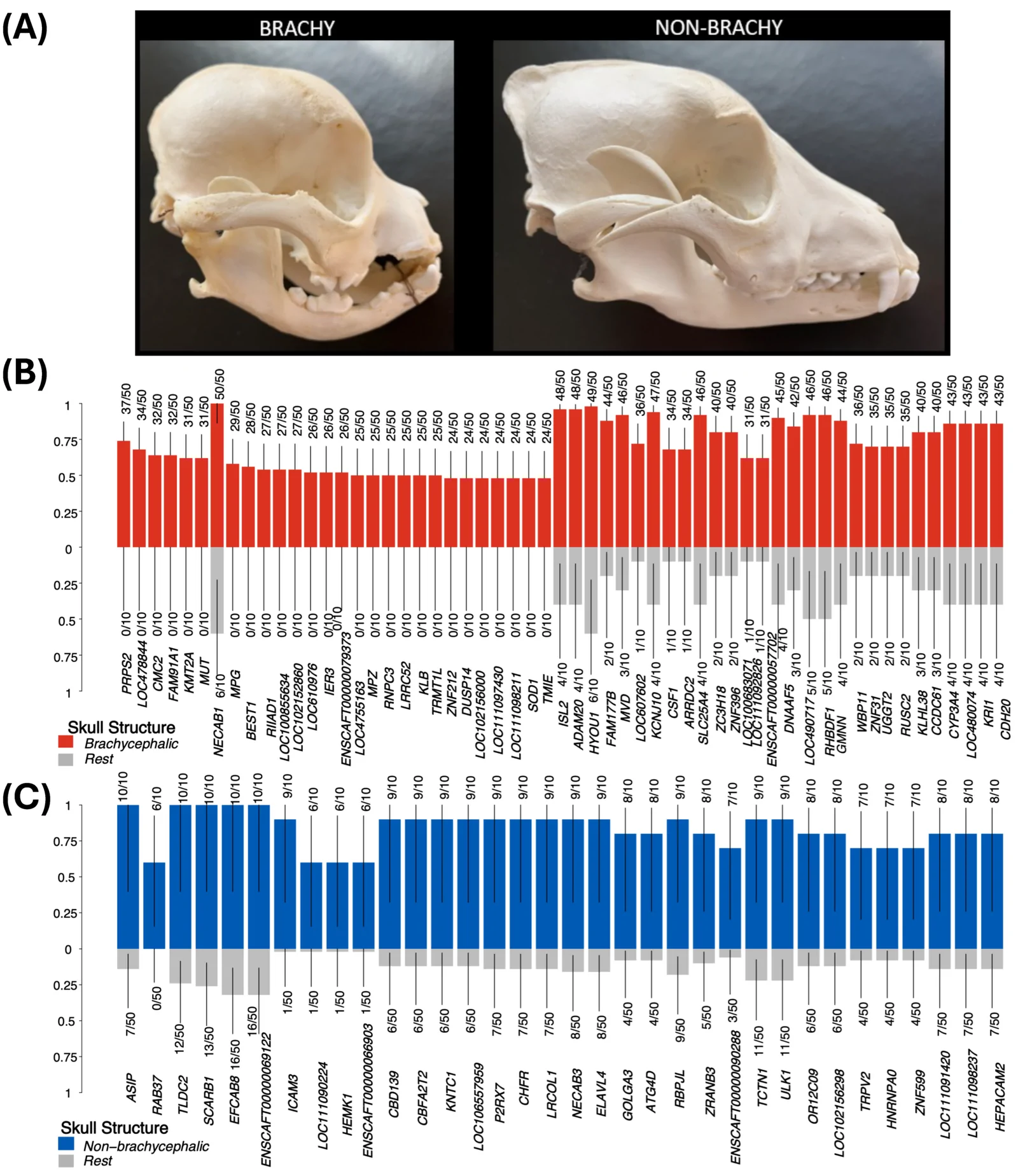
Prominent germline mutations in brachycephalic and non-brachycephalic dogs with glioma. (**A**) Representative photos of skulls of brachycephalic (**left**) breeds predisposed to glioma and non-brachycephalic (**right**) breeds predisposed to meningioma. In dogs, brachycephalic skull morphology is a significant predictor of brain tumor type [15,55]. Top germline mutated genes in (**B**) brachycephalic dogs with glioma and in (**C**) non-brachycephalic dogs with glioma. Enrichment plots of brachycephalic vs. non-brachycephalic (red columns), and of non-brachycephalic vs. brachycephalic (blue columns). Germline analysis was carried out on non-neoplastic tissues such as the liver and spleen.

Domesticated dogs exhibit two strikingly different craniofacial conformations: brachycephalic (short-skulled) and non-brachycephalic breeds (Figure 4). Most canine patients in this study were purebred brachycephalic dogs (71%, 56/79). To examine genomic differences associated with craniofacial morphology, gliomas originating in brachycephalic versus non-brachycephalic dogs were compared (Table 4). Mixed-breed dogs, which may include variable brachycephalic ancestry, were not included in either the brachycephalic or non-brachycephalic sub-groups.

Comparison of somatic (intra-tumoral) mutation profiles identified 118 non-synonymous (potentially cancer-causing) mutations that were significantly (*p* < 0.01) less common in brachycephalic compared to non-brachycephalic glioma. These included genes represented in the WNT pathway (*WNT3A*, *CAMK2D*), the cell-cycle pathway (*ESCO1*), the interferon-gamma IFNG pathway (10 genes including *PARP14* and *ACTA2*), and two known oncogenes (*EWSR1*, *NUP98*).

**Figure 5:**
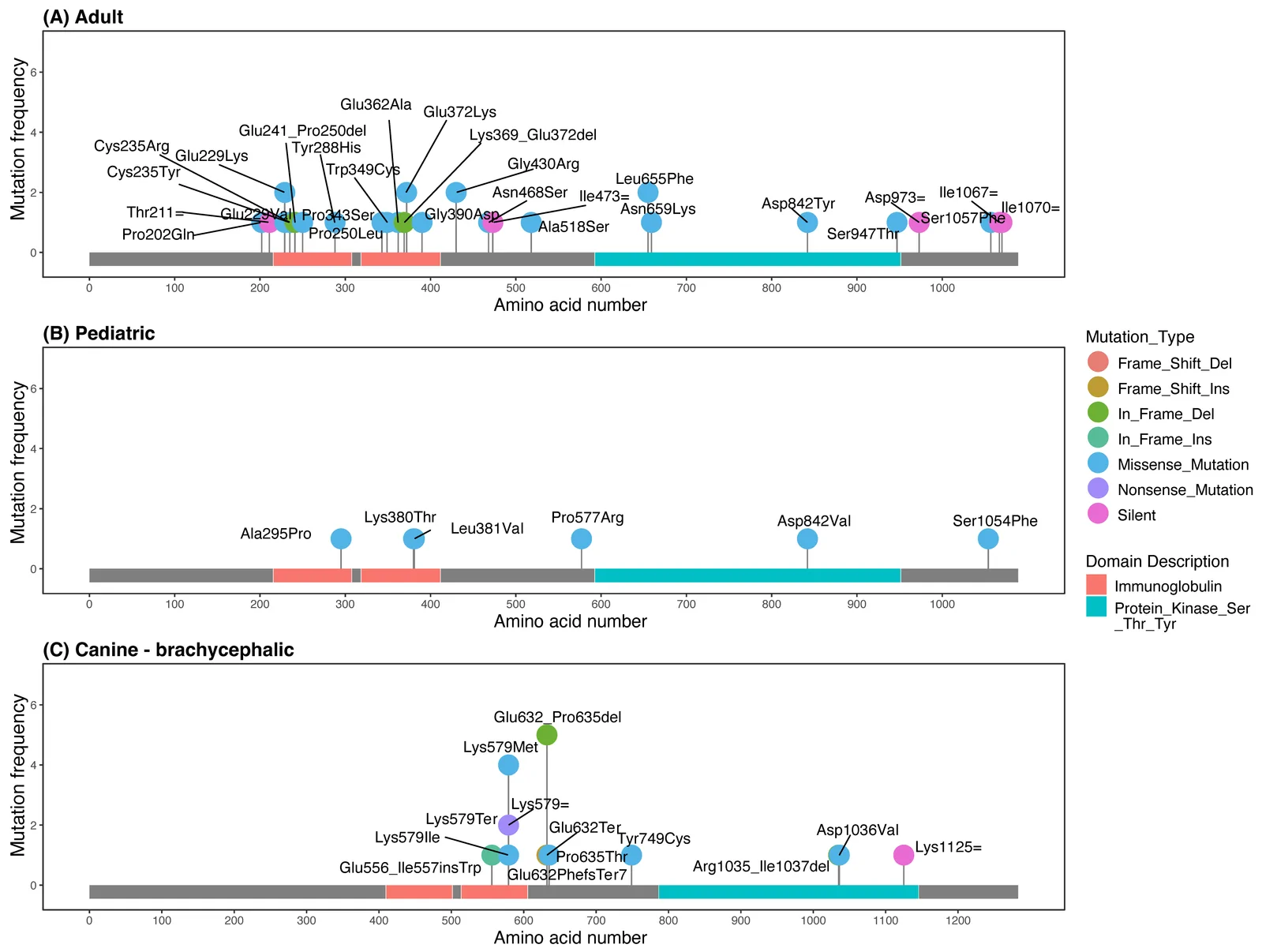
Non-synonymous PDGFRA mutations in canine and human glioma (low- and high-grade). Only mutations that lie in protein-coding regions of the gene are shown. Colors depict types of mutations. PDGFRA events were the most common mutations in canine vs. human glioma, and specifically in brachycephalic vs. non-brachycephalic canine glioma. (**A**) Adult glioma (22 mutations in 987 patients, a mean of 2.2 mutations/100 patients). (**B**) Pediatric glioma (five mutations in 118 patients, mean of 4.2 mutations/100 patients). (**C**) Brachycephalic dog glioma (19 mutations in 56 patients, mean of 33.9 mutations/100 patients). There were no mutations in 16 non-brachycephalic pure-bred dogs, and only 1 in 8 mixed-breed dogs.

In contrast, somatic alterations in *PDGFRA* were observed exclusively in brachycephalic gliomas. Sixteen of 56 (29%) brachycephalic tumors harbored non-synonymous *PDGFRA* mutations (including three tumors with two mutations each; a total of 19 protein-coding mutations) (Figure 5, Table 4). No somatic *PDGFRA* mutations were detected in non-brachycephalic purebred dogs (0/15, 0%; Bonferroni *p* < 0.05), and only one mutation was observed among mixed-breed dogs (1/8, 13%). By Firth’s penalized logistic regression, *PDGFRA* mutation was strongly associated with brachycephaly (adjusted odds ratio = 16.12, 95% CI: 2.0–2100, *p* = 0.0046). Copy number variation at *PDGFRA* showed a similar pattern: 9/56 (16%) brachycephalic gliomas harbored CNV compared to none in non-brachycephalic gliomas (0/15). This difference was not statistically significant after multiple testing correction (Bonferroni *p* = 0.565). Expression of *PDGRA*, an oligodendrocytic gene, was elevated 5.85-fold in canine glioma relative to normal brain (Table 3) and was higher yet again in brachycephalic relative to non-brachycephalic glioma (Table 4). Meanwhile, the astrocytic gene *GFAP* was under-expressed in brachycephalic glioma compared to non-brachycephalic (logFC −2.60, adjusted *p* = 0.0049) (Table 4). Interestingly, expression of PDGF-A, a ligand for the PDGFRA receptor, was significantly reduced in brachycephalic gliomas. Overall, *PDGFRA* alterations were enriched in brachycephalic dogs, with somatic *PDGFRA* mutations restricted to this group.

### 3.5. Germline Mutations in Brachycephalic and Non-Brachycephalic Cohorts of Canine Glioma Patients

Given the substantial differences in the incidence and molecular profile of brachycephalic glioma, we next examined germline (non-neoplastic) mutations. Data were available from the liver, spleen, heart, kidney, or unaffected brain of 50 brachycephalic and 10 non-brachycephalic canine cases.

Given the remarkable somatic differences between brachycephalic and non-brachycephalic gliomas, *PDGFRA* was examined. Despite applying the moderate-to-high level filter, germline variants in *PDGFRA* were common in both craniofacial groups (Table 4).

Many genes carried individual germline mutations at an increased incidence in one cohort or the other. Brachycephalic mutations were led by *PRPS2* (Figure 4B and Figure S3, Table 4). *PRPS2* carried 83 missense mutations in 37 brachycephalic dogs, despite not being mutated in any non-brachycephalic dogs, and was associated with the brachycephalic phenotype (adjusted odds ratio = 28.21, 95% CI: 3.5–1307.7, adjusted *p* = 0.026). Two variants (P299S and R304P) mapped within the conserved catalytic domain of *PRPS2*, while C331R, I333V, and H387R localized to the C-terminal region implicated in enzyme oligomerization and regulatory interactions. In the pathway analysis, germline mutations in TOR signaling were extremely common in both craniofacial cohorts (Table S9). Figure S4 shows mutational burden in selected pathways in the brachycephalic versus non-brachycephalic cohorts. In both cohorts, germline mutations were extremely common in multiple pathways.

### 3.6. Concurrent Pro- and Anti-Tumor Immune Signatures in Human and Canine Glioma

Cibersort and quanTIseq analysis disclosed an influx of anti-tumor inflammatory cells (neutrophils, M1 and CD4+ lymphocytes) across adult, pediatric, and canine glioma (Figure S5). The immune cell abundances should be interpreted as ortholog-converted, cross-species computational estimates rather than direct quantitative measurements, and should be used to identify relative immune patterns across cohorts and species. However, all 3 cohorts showed infiltration of pro-tumor immunosuppressive cells (M2 macrophages and T-regs) alongside marked reductions in key anti-tumor effector populations such as CD8^+^ and T follicular helper (Tfh) cells.

Macrophage composition shifted substantially, with the depletion of resting M0 cells and expansion of both M1 and M2 subsets. M2 macrophage elevations were significant in adult and canine cohorts, affecting LGG and HGG. Multiple CD4^+^ subsets expanded, including naïve CD4^+^ cells in LGG and memory CD4^+^ cells in HGG, whereas CD8^+^ and Tfh cells were consistently depleted across cohorts. Naïve B cells increased in adult and canine gliomas, while plasma B cell patterns differed between species.

Regulatory T cell (T-reg) estimates require methodological refinement. Cibersort yielded artifactually high T-reg levels in normal brain, which conflict with well-established human [96,97] and canine [1,17,42,98] data. Reanalysis with quanTIseq, which does not force cell-type assignment, confirmed near-zero T-regs in normal tissue with significant increases in adult LGG and HGG (Figure S6).

ssGSEA (Figure 3) demonstrated that the canine immuno-oncology gene panel (Canine IO [89]) was enriched across cohorts. Immune-enhancing genes [90] increased with grade in adult glioma. In dogs, LGG favored immune-enhancing pathways, and HGG favored immune-suppressive pathways [90].

Immune and stromal scores (Figure 3) were significantly elevated in adult and canine gliomas, reflecting increased immune and stromal content compared to the normal brain. Grade-level patterns varied across cohorts. Immune scores decreased with grade in canine and pediatric cases. Stromal scores increased with grade in canine and adult cases.

## 4. Discussion

### 4.1. Overview

GBM and other HGGs remain among the most lethal human cancers, with limited therapeutic progress despite extensive molecular characterization and clinical trials. A central challenge has been the lack of preclinical models that faithfully recapitulate not only the molecular drivers of disease but also the complex TME and its interactions with an intact host immune system [1,42]. Spontaneously arising gliomas in companion dogs have been increasingly used as a complementary translational model, yet the extent to which canine gliomas mirror human molecular, epigenetic, and immunological features has not been comprehensively established.

This study provides the first integrated comparison of the three canonical GBM oncogenic pathways—RTK/RAS/PI3K, p53, and Rb—across adult human, pediatric human, and canine glioma, along with an extensive evaluation of tumor methylation patterns, gene expression, and the immune composition of the TME. Importantly, the analysis extends beyond mutation and CNV to incorporate RNA sequencing–based differential gene expression and pathway-level activity, thereby offering a more complete representation of oncogenic signaling. In addition, this work uniquely interrogates molecular differences between brachycephalic and non-brachycephalic canine gliomas, providing insight into breed-associated tumorigenesis. Brachycephalic dogs have been bred to have progressively narrower upper airways over generations, possibly suggesting a complex interplay between anthropomorphic skull shape, systemic hypoxia, glioma risk, and intratumoral hypoxia.

Collectively, the findings demonstrate that human and canine gliomas differ in the initiation of genomic and epigenetic events but converge on a shared malignant phenotype characterized by pervasive dysregulation of canonical signaling pathways and a profoundly immunosuppressive TME. The importance of pet dogs in the study of human cancers is well established [4,5,6,8,99,100]. The current results strongly support the continued use of canine glioma as a biologically relevant model for human glioma, especially in immunotherapy development.

### 4.2. Canonical Pathway Dysregulation Across Species

The three canonical pathways of human GBM are near-universally disrupted in adult GBM and form the backbone of current molecular models of gliomagenesis [94]. Traditionally, “abnormal signaling” has been defined by mutation and CNV; however, this approach may underestimate pathway activity when transcriptional dysregulation is not considered. By incorporating RNA-seq-based DEG analysis, this study reveals that abnormalities within these pathways are even more extensive, including in canine HGG (Figure 1B).

In adults and dogs, the frequency of canonical pathway disruption was associated with tumor grade (Table 2). Abnormalities in all three pathways were largely restricted to HGG. Importantly, when tumors were stratified by grade rather than species, molecular differences between adult human and canine tumors were reduced, indicating that glioma grade exerts a stronger influence on pathway dysregulation than host species.

In canine HGG, inclusion of DEGs revealed widespread aberrant signaling across all three pathways (Figure 1B). This finding underscores the importance of transcriptional dysregulation in malignant signaling. Molecular complexity may be underestimated when relying solely on mutation-centric frameworks.

### 4.3. RTK/RAS/PI3K Pathway, and the Central Role of PDGFRA in Brachycephalic Glioma

Among the canonical pathways, the RTK-PI3K-mTOR axis emerged as particularly prominent across species (Figure 1 and Figure 3). Other similarities included loss of the tumor suppressor genes *PTEN* and *NF1* (Figure 1A). In canine glioma, *PDGFRA* rather than *EGFR* dominated activation of the RTK pathway (Table 3).

*PDGFRA* represented one of the study’s most striking findings. It was mutated in approximately one-quarter of all canine gliomas and in one-third of canine HGG (Figure 1A), compared with much lower rates in humans. Canine *PDGFRA* showed marked copy number gain and significant overexpression at the transcriptomic level. This finding is supported by a study using immunophenotyping and the alternative canine grading schema, revealing *PDGFRA* expression in 14% of grade II gliomas and 43% of GBMs [101]. When combined with overexpression of *PDGFR-beta* (Figure 1B), these alterations suggest ligand-independent activation of the RTK pathway in canine glioma.

Notably, *PDGFRA* alterations were even more pronounced in brachycephalic dogs, where somatic mutations were common, with some tumors harboring multiple mutations (Figure 5). This enrichment was observed despite the presence of *PDGFRA* germline mutations in both brachycephalic and non-brachycephalic dogs, indicating that intra-tumoral selection rather than inherited variation may drive *PDGFRA* activation in brachycephalic glioma.

The biological significance of *PDGFRA* activation is reinforced by its identification as an early clonal event in adult, pediatric, and canine gliomas [3,102]. Together, these data position *PDGFRA* as a conserved and critical driver of glioma biology across species, albeit with species- and grade-specific patterns of activation.

Significant downregulation of IGF, paired with logFC > 2.0 upregulation of the IGF receptor in canine HGG (Figure 1B), is also interesting. IGF-I antisense therapy has had initial success in human patients [103], representing another therapeutic avenue that could be developed in canine patients, given the conserved post-receptor RAS-PI3K signaling between species.

### 4.4. p53 Pathway: Divergent Genotypes, Convergent Tumor Suppressor Inactivation

The p53 pathway displayed both shared and divergent features across species. While *TP53* mutations were concentrated in human glioma (Figure 1A), TP53 was significantly overexpressed in both adult and canine tumors (Table 3). These findings suggest fundamentally different mechanisms of p53 pathway alteration. In adult glioma, *TP53* overexpression likely reflects accumulation of mutant, non-functional p53 tetramers, a well-established phenomenon in human cancer. In contrast, the rarity of canine *TP53* mutations implies that overexpression (Figure 1B) likely represents a physiological response to oncogenic stress, with wild-type p53 attempting—ultimately unsuccessfully—to restrain tumor progression.

Amplification of p53 inhibitors (*MDM2* and *MDM4*), intensified by *CDKN2A/B* deletion (Figure 1A), provides shared mechanisms for attempted p53 suppression. Thus, although initiating events differ, gliomas in both species attempt inactivation of p53 tumor suppressor function, illustrating convergent pathway-level outcomes despite divergent genotypes.

### 4.5. Rb Pathway and Cell Cycle Dysregulation

The Rb pathway showed substantial overlap between adult human and canine glioma. At the pathway level, both species demonstrated clear loss of cell cycle control (frequent deletion of *CDKN2A/B*, cyclin amplification, partial loss of CDKN2C, and occasional *RB1* mutation; Figure 1A). These alterations collectively promote unchecked cell cycle progression, a hallmark of high-grade malignancy.

Interestingly, expression of Rb1 itself differed between species, with underexpression in canine glioma and overexpression in adult glioma (Table 3). These opposing transcriptional changes may reflect different compensatory responses to upstream pathway disruption rather than differences in pathway function.

### 4.6. ID1 as an Upstream Integrator of Malignant Signaling

*ID1* emerged as a notable upstream regulator (Table 3). ID1 promotes malignancy by enhancing RTK signaling through increased EGF secretion; it has been implicated in glioma invasiveness, chemoresistance, aberrant vasculature, and resistance to anti-angiogenic therapy [104,105,106].

This demonstration of *ID1* overexpression in human and canine primary glioma tissue extends prior findings in cell lines and xenografts [104,105,106], reinforcing its relevance as a therapeutic target. Given its role in multiple oncogenic processes, *ID1* could represent a cross-species vulnerability.

### 4.7. Mutation Burden, Tumor Grade, and Pathway-Level Perspective

Canine glioma exhibits 0.34 coding mutations per megabase, matching pediatric glioma (0.25–0.34, depending on subtype) rather than adult IDHmut glioma (0.77) or IDHwt glioma (1.67) [3]. However, when analysis was restricted to the three canonical pathways, canine HGG approached adult HGG in the frequency of mutations, especially *PDGFRA*, *CDKN2A/B* and cyclins (Figure 1A). Abnormal pathway signaling occurs on a spectrum, from canine and human LGG to adult GBM [94,107] (Table 2). The apparent mutation rate in HGG may be influenced by the overrepresentation of grade 4 GBM in adults (62% of gliomas [108]), compared with grade 3 gliomas in dogs (55–59%) [8,53].

The RTK pathway and its effector arms (PI3K/mTOR, RAS/MAPK) were assessed in overlapping analyses. Only the PI3K sub-pathway was mutated more commonly in canine LGG than HGG (Figure 2B). This is consistent with insights that PI3K/mTOR signaling occurs most often in adult LGG, predicts survival, and is a potential pediatric LGG treatment target [109,110]. There was robust activation of the parent PI3K/AKT/mTOR pathway in canine and pediatric LGG (Figure 3). Germline mutations in TOR signaling were near-universal in dogs (Table S5). mTOR combats immunosuppression through multiplex effects: TGFβ and IL-1 impact T cells, microglia, and macrophages, upregulating MHC expression and antigen presentation and preventing macrophage polarization [111]. Activation of the MAPK sub-pathway was strongest in canine HGG (Figure 3). MAPK is an effector arm of the RTK/RAS pathway that regulates cell proliferation and cell death [112]. Seeing more consistent elevation in canine HGG than adult HGG is striking; with *ID1*, it reveals critical activations upstream and downstream of the canonical pathways.

These findings highlight the limitations of mutation burden as a sole metric of biological aggressiveness and reinforce the value of pathway-level analyses. Across species, individual gene mutations were uncommon, and yet dysregulation at the pathway level was nearly universal in HGG. This observation supports a model in which glioma evolution is driven by selective pressure to disrupt signaling networks rather than specific genes. Further, heterogeneity between patients was a major similarity between species. For example, some human gliomas carry the PDGFRA mutation, exemplifying canine glioma, while occasional canine gliomas bear the IDH mutation that has become the hallmark of human LGG.

### 4.8. Epigenetic Divergence: IDH and DNA Methylation

A major distinction between adult glioma and both pediatric and canine glioma lies in DNA methylation patterns (Table 3). Adult gliomas were predominantly hypermethylated (high beta values) as expected with gain-of-function *IDH1* mutations, whereas pediatric and canine gliomas were largely hypomethylated (low beta values).

IDH1/2 mutations were widespread in adult LGG yet rare in the other cohorts (Table S8). While IDH mutations are strongly associated with hypermethylation, when specifically compared to other IDH1/2 mutations, IDH1^R132H^ gliomas have lower DNA methylation levels and a worse prognosis [113]. IDHwt status is now a defining feature of human GBM [44]. We found that in canine gliomas, IDH1^R132C^ is the most common non-canonical IDH1 [3] substitution and, like IDH1^R132H^ in human gliomas [114,115,116,117,118], disrupts the conserved active-site arginine required for substrate coordination (Figure S2), resulting in comparable neomorphic 2-hydroxyglutarate-producing activity [95]. Its relative frequency, however, varies considerably by tumor context: in humans, IDH1^R132C^ is disproportionately represented among infratentorial astrocytomas, Li-Fraumeni syndrome-associated gliomas [119], and AML, likely reflecting context-dependent selection [120] rather than uniform biological equivalence across tumor types. Notably, IDH1^R132H^ and IDH1^R132C^ arise from the same spontaneous CpG-to-TpG deamination event at the R132 codon on opposite DNA strands [121], suggesting the tissue-specific skew reflects differential selective pressure, including co-occurrence with TP53/ATRX alterations [121], rather than differences in mutational probability per se.

Corresponding to the strong overexpression of *IDH1* in adults, *MGMT* and PD-1 were hypermethylated in adults but hypomethylated in dogs (Table 3). These differences have direct clinical implications. *MGMT* methylation predicts temozolomide chemotherapy responsiveness. *PD-1* is expressed by both effector and regulatory T cells, where the balance of its expression can predict the efficacy of immune checkpoint blockade [122].

Despite these epigenetic differences, downstream oncogenic signaling and the immune landscape converged across species, suggesting that methylation status influences the route to malignancy and an immune cold TME [3,123], rather than the final phenotype.

### 4.9. Immune Landscape and the Immunosuppressive TME

One of the most significant contributions of this study is the detailed comparison of the immune TME across species (Figure 3 and Figure S5, Table 5). Adult and canine gliomas exhibited a complex, predominantly immune-cold microenvironment characterized by infiltration of inflammatory cells (neutrophils, M1 macrophages, CD4 lymphocytes), increased immunosuppressive cells (T-regs, M2 macrophages), and subsequent depletion of key antitumor effector populations (CD8 lymphocytes, Tfh lymphocytes) (Figure S5). These many changes were significant in adult HGG; most were also significant in canine HGG. The current data are derived from RNA-seq, but they are supported by direct cell counts showing neutrophils, macrophages, and T-regs within canine glioma [3], with increased M1 and M2 macrophages in HGG compared to LGG [98].

Infiltration of inflammatory cells, such as neutrophils and M1 macrophages, is a physiological response to neoplasia and tumor necrosis [3]. Changes in CD4 subsets (naïve, memory resting, and memory activated) were seen across cohorts. T cell lymphopenia is a well-documented feature of human GBM [124], where CD4^+^ T-helper cells are sequestered in the bone marrow [125]. T-helper cells kill tumor cells directly and indirectly [97]. Intriguingly, bone marrow T cell accumulation was observed when mice were intracranially implanted with GBM, but not when implanted subcutaneously [125]. The matching intracranial changes in CD4 subsets between adult and canine glioma build the canine model.

Regulatory T cells (T-regs) are rarely observed in normal human and canine brains [1,17,42,96,97,98,126]. Utilizing IHC, none were detected in the control canine brain or the non-neoplastic brain of canine glioma patients [1]. QuanTIseq analysis (Figure S6) demonstrated scant T-regs in the normal brain, with elevated levels in adult and canine glioma, supporting immune evasion. These data are consistent with flow cytometry counts of T-regs in canine glioma tissue and peripheral blood [17], and the trafficking of T-regs towards canine HGG [126]. In adults, T-regs were highest in HGG (Figure S6). In a canine study, T-regs were similarly highest in high-grade astrocytoma [1]. Meanwhile, macrophage polarization favored the M2 phenotype across all three cohorts, further supporting a protumor, anti-inflammatory milieu. Polarization of the M1:M2 ratio towards M2 macrophages occurs in human GBM, decreasing immune system effectiveness [127].

CD8 T cells, which are central to antitumor immunity and current immunotherapy strategies [17,97,128], were reduced relative to true-normal brain control tissue. These cells have been reported to be increased in HGG, but often in comparison to tumor-adjacent brain, matched peripheral blood, or low-grade glioma [17,129,130]. Additionally, some tumor-infiltrating lymphocytes in GBM are “exhausted” [130]. There were also depletions of Tfh lymphocytes. These cells can sustain exhausted T cells and restore anti-PD-L1/PD-1 efficacy [97,131], and their elevation is associated with an improved prognosis [132]. Their marked depression adds to the evidence of a shared immunosuppressive TME.

Plasma cells were decreased in adult glioma, yet increased in canine glioma. These results might relate to higher GBM incidence versus oligodendroglioma in the adult versus canine cohorts (Figure S7). In humans, plasma cells are a leading infiltrate in oligodendroglioma [133], while B cells are depressed in GBM [134].

Alterations in immune checkpoint biology further reinforced this immunosuppressive phenotype. The binding of PD-L1 (on tumor cells) to PD-1 (on T cells) is critical for establishing an immunosuppressive TME. *PD-L1* was preserved in both species (hypomethylated, minimal mutation) and overexpressed in canine glioma (Table 3). *PD-1* was hypomethylated in dogs and overexpressed in adults. This suggests a dynamic selection for intact immune checkpoint pathways in glioma evolution. An ssGSEA analysis revealed activation of immune suppressive genes in all three cohorts (Figure 3B). While this was prominent in adult HGG, a larger selection of canine gliomas revealed individuals with very strong enhancement (Figure 3A).

The matching immune cell aberrations might be explained by chemokine biology. *HLA-G* expression, which downregulates chemokine receptors on lymphocytes and functions in GBM immune escape [135], was increased as expected (Table 3). Surprisingly, adults and dogs showed decreased *CXCL12* expression. This chemokine is secreted by tumor cells and pericytes, binds receptors in tumor cells and macrophages, and encourages tumor cell–macrophage co-invasion [136].

Cross-species immune derangement was further confirmed by JNK/stress-activated activation in all three cohorts (Figure 3), a pathway involved in both chronic inflammation and neurodegeneration [137]. While inflammation can impede tumor progression, signaling in the JNK pathway leads to brain infiltration via matrix metalloproteinases and neuronal degeneration through Wg depletion [138], thereby increasing symptomology. The Canine IO panel, a collection of both enhancing and suppressing immune genes [90], was also elevated across cohorts. The comparable Nanostring IO (immuno-oncologic profiling) panel is activated in pediatric LGG and prognostically relevant [123].

An alternative method of studying the TME (Figure 3C) showed significant infiltration of immune cells (irrespective of pro- or anti-inflammatory nature). There was also a significant presence of stromal cells. The ESTIMATE scores, derived from these immune and stromal scores, were correspondingly elevated. Higher scores reflect “impure” tumors infiltrated by immune and stromal cells. Canine glioma occupied a middle ground—stromal scores were higher in HGG (matching adult), but immune scores were higher in LGG (matching pediatric).

Overall, these data add considerable weight to the position that the canine and human HGG TME is “immune cold” [3,33,123], though it should be noted that because sample acquisition and treatment exposure are highly confounded with cohort identity, their independent effects cannot be reliably estimated. Therefore, observed TME differences may reflect both biological differences and differences in tissue acquisition or treatment history. A potential cause of the occasional differences in immune characterization of the three cohorts is administration of glucocorticoids prior to glioma sampling (see Section 4.12 below).

### 4.10. Brachycephalic Glioma and Proneural-Subtype Parallels

Brachycephalic dogs have a strongly increased glioma risk [15,56]. Their craniofacial conformation also results in brachycephalic obstructive airway syndrome (narrow upper airways with redundant soft tissues). The French bulldog breed (five cases in our study) presents extreme brachycephaly and is chronically hypoxic [57]. Hypoxia generates transcriptomic changes in glioma and might contribute to the development of HGG in French bulldogs and other brachycephalic breeds [57,139,140]. We therefore performed the first extensive molecular analysis of brachycephalic versus non-brachycephalic canine glioma (Figure 3 and Figure 4, Table 4 and Table S9).

Brachycephalic canine gliomas emerged as a molecularly distinct subset, with reduced *GFAP* expression and marked enrichment for *PDGFRA* (mutation, high amplification, overexpression), consistent with an oligodendroglial phenotype (Table 4). Diagnostic canine and human GFAP IHC is usually negative in oligodendrogliomas and positive in astrocytomas/GBM [49,141]. However, in human GBM, negative GFAP IHC is associated with reduced survival [141].

*PDGFRA* is an oligodendrocyte development gene functioning in oligodendrocyte progenitor cells [107,142]. Certain brachycephalic breeds, including the French bulldog, have an elevated rate of oligodendroglioma over astrocytoma [6,15]. In current cohorts, 16 brachycephalic gliomas carried 19 non-synonymous *PDGFRA* mutations (Figure 5), more than all non-brachycephalic (0 mutations) and mixed-breed cases (1 mutation). PDGFRA is activated by dimeric isoforms of PDGF-A and PDGF-B (PDGF-AA, PDGF-AB, PDGF-BB). The brachycephalic underexpression of *PDGF-A* may be a response to PDGFRA enrichment. These features closely parallel the Proneural subtype of human GBM, characterized by PDGFRA activation and abnormal EGFR signaling, alongside *IDH1* events [107]. Similarly, human oligodendroglioma carries the Proneural signature [143]. The restriction of somatic *PDGFRA* mutations to brachycephalic dogs suggests that genetic background may influence the likelihood of selecting for intratumoral *PDGFRA* alterations. Dog breeds exhibit strong population structure and extended linkage disequilibrium due to founder effects and selective breeding, which can shape both germline variation and somatic mutational landscapes. The high prevalence of germline *PDGFRA* variants in brachycephalic dogs may reflect breed-associated haplotypes at or near this locus, potentially altering local genomic context or pathway activity. In this setting, tumors arising in brachycephalic dogs may be more likely to acquire additional somatic mutations in *PDGFRA*, either because of underlying genetic susceptibility or because such alterations confer a selective advantage in that background. While the functional consequences of these variants remain to be determined, these findings suggest that perturbation of PDGF-PDGFRA signaling is particularly relevant in brachycephalic dogs and underscore the importance of considering breed structure when interpreting somatic mutation patterns in canine cohorts.

Germline (inheritable) differences (Figure 4, Table S9) accompanied the somatic (intra-tumoral) alterations. Notably, *PRPS2* was mutated in the majority of brachycephalic dogs but in none of the non-brachycephalic dogs (Table 4). *PRPS1* and *PRPS2* expression is decreased by hypoxia and endoplasmic reticulum stress [144], offering a fascinating insight into the possible etiology of glioma in these chronically hypoxic brachycephalic patients. *PRPS2* encodes Phosphoribosyl Pyrophosphate Synthetase 2, an enzyme that catalyzes the formation of phosphoribosyl pyrophosphate (PRPP), a key precursor required for the biosynthesis of purine, pyrimidine, and pyridine nucleotides [145]. *PRPS2* has elevated expression in cancer and oncogenic functionality [146,147,148]. The distribution of *PRPS2* variants suggests that a subset of mutations may directly affect catalytic activity, whereas others may influence PRPS2 function through structural or regulatory mechanisms affecting enzyme assembly.

Chronic hypoxia associated with brachycephalic obstructive airway syndrome might potentially shape somatic and germline mutation patterns. These manifold findings not only enhance understanding of breed-associated glioma risk but also distinguish brachycephalic gliomas as a particularly informative subgroup for translational research.

The lack of statistically significant differential pathway enrichment (IPA, GO, and Reactome) between brachycephalic and non-brachycephalic samples suggests that the overall activation of each pathway is similar despite substantial differences in individual genes, such as PDGFRA. Alternatively, the lack of statistically significant results could be due to a small sample size, particularly in non-brachycephalic breeds.

### 4.11. Canine Glioma as a Translational Model

Adult, pediatric, and canine gliomas are “the same, but different”. In terms of CNV (aneuploidy), overall mutation rate, absent IDH mutation, and hypomethylation, canine glioma best matches pediatric. However, it is adults and dogs who share the propensity for HGG [6]. Despite differences in mutation burden, methylation, and histology, canine HGG shares extensive molecular and immunological overlap with adult HGG (Table 5) [5,17,21,22,26,53,54]. Comparative pathology boards, comprising human and canine pathologists, are providing insight into the diagnosis and pathogenesis of glioma [45].

This human–canine convergence supports the concept of convergent evolution, in which tumors arising from distinct genetic starting points ultimately develop similar malignant phenotypes under shared selective pressures imposed by the brain microenvironment and the immune system [3]. Histologically, many canine gliomas resemble malignant oligodendrocytes, while many human gliomas resemble astrocytes, yet there is growing recognition of their molecular similarities. Further, molecular characterization is beginning to supersede histology [44,149]. The predominance of high-grade oligodendroglioma in dogs should be interpreted in light of the finding that human pathologists are significantly less likely than veterinary pathologists to report infiltration and necrosis, and to diagnose oligodendroglioma [45].

Canine glioma might occupy a middle ground between adult and pediatric glioma. Arguably, the question shouldn’t be whether canine glioma most closely resembles adult or pediatric glioma, but rather which is the best pre-human model for developing each specific therapy. Canine gliomas develop spontaneously in immunocompetent hosts, exhibit natural tumor heterogeneity, and respond to therapy in clinically meaningful ways (e.g., much shorter post-operative survival in HGG [150])—features that are difficult to model in rodents. The many parallels between the human and canine TME strongly support the extensive usage of canine glioma in immunotherapy development, including vaccines, oncolytic viruses, and other immune-modulating agents, underscoring its translational value [8,20,26,27,28,29,30,33,39,150,151,152,153].

Finally, given their large brain size, canine glioma patients have been used to develop intra-tumoral delivery of viruses, cytotoxins, chemotherapy, and laser interstitial thermal therapy [23,24,34,35,36,38,39,151,153].

### 4.12. Limitations

Our Cibersort and QuanTIseq results were not confirmed with IHC or flow cytometry. These techniques use gene expression signatures to infer cell numbers, rather than directly count cells. These gene expression signatures were primarily defined for the human genome and reused for cell quantification in dogs using orthologous genes. There is admittedly less annotation of the canine genome, and canine expression data may not be available for all the genes within a given molecular signature. These factors could explain, for example, the strong monocyte signatures observed in normal canine brain. While many of our results were highly consistent with prior reports of human and canine glioma, findings such as CD8 reductions require corroboration by other techniques.

It is worth considering the impact of any therapy prior to glioma tissue harvesting, as this differed across the three cohorts. Adult glioma tissue is typically harvested at surgery after initiating glucocorticoid therapy [94]. Steroids act upon gene expression, the vasculature, and the immune system to reduce peritumoral edema and intracranial pressure. Pediatric glioma tissue was harvested at autopsy, which typically follows mutagenic therapies (radiation therapy, chemotherapy) and steroids [154]. Some canine patients received steroids, with a subset receiving mutagenic therapies, prior to euthanasia and glioma sampling. Some of the apparent differences between cohorts in both the immune TME and genomic analyses could therefore arise from differences in therapies administered prior to glioma tissue collection. Euthanasia without any treatment, commonplace in canine glioma [6], should be leveraged to study the TME of truly treatment-naïve tissue. Unfortunately, the clinical metadata were not uniformly available across all datasets. As a result, formal sensitivity analyses evaluating the effects of treatment exposure and tissue acquisition variables could not be performed, although these factors are likely to influence the TME and gene expression data. Future studies with harmonized clinical annotations and prospectively collected samples will be needed to more fully disentangle biological differences from potential treatment- and collection-related effects.

The WHO system for classifying and grading human glioma has evolved. Future studies should take advantage of the latest classification system. However, vitally useful tumor banks have been constructed using prior WHO systems, including those leveraged in our study. The human cases in our study were classified under the 4th edition [47] rather than the current 5th edition [44]. Tumor type (astrocytoma, oligodendroglioma, glioblastoma) can be reasonably reassigned using IDH mutation status and copy-number profile (1p/19q co-deletion). As many features are unchanged between the 4th and 5th editions, most cases would have the same diagnosis. Notable exceptions include the discontinuation of oligoastrocytoma (still known as “undefined glioma” in dogs) and the increased weight given to genetic features, including IDHwt status, which has become a defining feature of human GBM independent of histology. Reassigning tumor grade, which underlies our LGG/HGG grouping, is considerably more difficult: the 5th edition grade 2/3 distinction relies on histological mitotic activity and cytological anaplasia, and is not fully resolvable from the TCGA data available to us (e.g., chromosome +7/−10 and TERT promoter status are missing for a substantial fraction of cases).

It seems unlikely that it would be advantageous to use the 5th human edition to diagnose canine glioma. IDH status does not seem to distinguish between canine gliomas—nearly all are IDHwt. In fact, because most canine gliomas display necrosis or microvascular proliferation [17,26,51] and are IDHwt [3,18,155,156], they would hypothetically be classified as GBM if applying the 5th edition. Instead, the CBTC canine glioma classification [6] is functional, co-created by veterinary and physician pathologists, was designed to support translational studies, has moderate inter-rater agreement, provides a simple grade (LGG or HGG), and has been widely adopted.

Our canine glioma dataset overlaps substantially with that published by Amin et al. [3]. Consistent with their findings, our analyses confirm the cross-species molecular similarity between canine and pediatric glioma. The present study, therefore, focused on novel additions: canonical GBM pathway analyses, enhanced TME analyses, and brachycephalic versus non-brachycephalic glioma comparisons, including PDGFRA alterations and germline mutations. Also, canine glioma was compared to true normals within this study. However, the number of these controls was limited, and they were not matched by breed and age to the canine glioma cohort. Normal pediatric RNA-seq data were entirely unavailable, preventing key analyses such as gene expression.

## 5. Conclusions

In summary, this study demonstrates that canine glioma, particularly high-grade disease, is molecularly and immunologically aligned with human glioma at the pathway and phenotype level, despite divergent initiating genotypes and epigenetic landscapes. Brachycephalic canine glioma particularly aligns with proneural adult GBM. The shared dysregulation of canonical pathways and the conserved immunosuppressive TME provide a strong biological rationale for continued use of canine glioma as a translational model, especially for immunotherapy development.

By moving beyond gene-centric comparisons to pathway- and phenotype-level analyses, this work reframes canine glioma not as an imperfect surrogate but as a complementary system that captures critical aspects of human disease poorly modeled elsewhere.

The ICDC website (https://caninecommons.cancer.gov/, accessed on 5 August 2020) provides complete genomic data from canine glioma, osteosarcoma, lymphoma, and bladder cancer cases as a free resource, and should be harnessed to support the translational study of these cancers.

## Figures and Tables

**Table 1:** Number of glioma tumor samples and true normal brain tissue samples. HGG denotes high-grade gliomas (grades 3 and 4). LGG denotes low-grade glioma (grades 1 and 2). GBM denotes glioblastoma. Astro denotes astrocytomas. Oligo denotes oligodendrogliomas. Oligo–astro denotes oligoastrocytomas. Glioblastomas were included within astrocytomas. In dogs, oligoastrocytoma is also known as an undefined glioma.

	Total Gliomas	LGG	HGG	Astro/GBM	Oligo	Oligo–Astro	Normal
Adult	1035	216 (21%)	752 (73%)	680 (66%)	174 (17%)	124 (11%)	464
Pediatric	258	193 (74%)	63 (24%)	258 (100%)	0 (0%)	0 (0%)	0
Canine	81	16 (20%)	65 (80%)	30 (37%)	45 (56%)	6 (7%)	12

**Table 2:** Comparison of canonical oncogenic pathway abnormal signaling in low-grade glioma (LGG) and high-grade glioma (HGG) with reported values for adult glioblastoma (GBM). Copy number variation (high amplification with ≥5 copies) and mutations were considered abnormal signaling. High-grade gliomas (grades 3 and 4) are shown in red, and low-grade gliomas are shown in blue. Glioblastoma (GBM) is a grade 4 astrocytoma. The percentage of cases with at least 1 pathways and all 3 pathways affected are shown in bold.

Pathways	15 Canine LGG	64 Canine HGG	214 Adult LGG	745 Adult HGG	GBM *Nature *2008 [94]
RTK/RAS/PI(3)K	47%	72%	53%	78%	88%
p53	20%	47%	50%	74%	87%
Rb	27%	53%	49%	75%	78%
**At least 1 pathway**	**53%**	**83%**	**78%**	**92%**	**95%**
**All 3 pathways**	**20%**	**34%**	**26%**	**58%**	**74%**

**Table 4:** Differences between gliomas in brachycephalic and non-brachycephalic purebred dogs (low-grade and high-grade combined). A positive RNA-seq log fold-change (logFC) value implies increased expression in brachycephalic compared to non-brachycephalic cases.

	Brachycephalic (n = 56)	Non-Brachycephalic (n = 15)
**PDGFRA** Somatic mutations (protein-coding only)	19 mutations, affecting 16 dogs	0/15
Somatic CNV	9/56 All high amplification	0/15
Somatic RNA-seq LogFC	+3.18 (adjusted *p* = 0.037)	
Germline mutations (mod/high impact only)	21 missense and 14 other mutations, affecting 19 dogs	7 missense and 1 frame shift insertion, affecting 5 dogs
**PDGF-A** Somatic RNA-seq LogFC	−1.63 (adjusted *p* = 0.048)	
**GFAP** Somatic mutations (protein-coding only)	0 mutations	2 mutations, affecting 2 dogs
Somatic CNV	0/56	0/15
Somatic RNA-seq LogFC	−2.60 (adjusted *p* = 0.0049)	
Germline mutations (mod/high impact only)	34 missense mutations, affecting 24 dogs	6 missense and 1 frameshift insertion, affecting 4 dogs
**PRPS2** Germline mutations	83 missense mutations, affecting 37 dogs	0/15

**Table 5:** Comparison of pediatric, canine, and adult glioma data—a summary. Significant and other substantial findings are highlighted in bold and italic.

Feature	Pediatric	Canine	Adult
Common Grades	LGG	HGG	***Grade 4***
Abnormal Signaling in the Three Pathways	***High***		
IDH Mutation	Rare		***Common***
Methylation	Hypomethylated		***Hypermethylated***
PDGF-A—PDGFRA Aberrations	Low	***High***	Intermediate
Aneuploidy (CNV) (Amin et al., Cancer Cell)	***High***		***High (IDHwt***) Low (IDHmut)
Inflammatory Cells (neutrophils, M1, CD4)	Further study required	***High***	
Immunosuppressive Cells (T-regs, M2)		***High***	
Anti-Tumor Effector Cells (CD8, Tfh)		***Low***	

## Data Availability

The RNA sequencing data for canine normal brain tissue samples generated in this study have been deposited in the Gene Expression Omnibus (GEO) under accession number GSE330017.

## Supplementary Materials

The following supporting information can be downloaded at https://www.mdpi.com/article/10.3390/curroncol33100592/s1. Table S1: Sample summary; Table S2: Canine samples metadata table; Table S3: Adult glioma (TCGA) samples metadata table; Table S4: Pediatric (KidsFirst) samples metadata table; Table S5: Adult normal (GTEx) samples metadata table; Table S6: Pathway gene details of Figure 1A; Table S7: Pathway gene details of Figure 1B; Table S8: IDH1 mutation status of glioma cases; Table S9: Differential germline mutations: brachycephalic and non-brachycephalic dogs; Figure S1: Copy number variation in genes of the 3 GBM canonical pathways (PI3K/MAPK, Rb1 and p53) in canine glioma (low- and high-grade); Figure S2: Multiple sequence alignment of wild-type and mutant IDH sequences (human and canine), highlighting position 132; Figure S3: 87 non-synonymous germline mutations in PRPS2, identified in 37 brachycephalic dogs; Figure S4: Germline mutational burden within oncogenic signaling pathways in brachycephalic (red) and non-brachycephalic (blue) dogs with glioma; Figure S5: Deconvolution of immune signatures computed by Cibersort [82]; Figure S6: Deconvolution of Treg signatures computed by quanTIseq [83]; Figure S7: Plasma cell infiltration in adult oligodendroglioma versus glioblastoma, and in canine oligodendroglioma versus high-grade astrocytoma.

## Abbreviations

The following abbreviations are used in this manuscript:

TME	Tumor Microenvironment
Indels	Insertion–Deletion Events
SNPs	Single Nucleotide Polymorphisms
GMB	Glioblastoma
WHO	World Health Organization
IDHwt	IDH-Wildtype
IDHmut	IDH-Mutated
IHC	Immunohistochemistry
CBTC	Comparative Brain Tumor Consortium
LGG	Low-Grade Glioma
HGG	High-Grade Glioma
GDC	Genomic Data Commons
CGC	Cancer Genomics Cloud
ICDC	Integrated Canine Data Commons
GTEx	Genotype-Tissue Expression Portal
CN	Copy Number
DMG	Differentially Mutated Gene
DEG	Differentially Expressed Gene
TPM	Transcript Per Million
RNA-seq	RNA Sequencing
GO	Gene Ontology
GSEA	Gene Set Enrichment Analysis
CNV	Copy Number Variation
ssGSEA	Single-Sample Gene Set Enrichment Analysis
Tfh	T Follicular Helper
T-reg	Regulatory T Cell
CRDC	Cancer Research Data Commons
Kids First	Gabriella Miller Kids First Pediatric Research Program
