# Supplementary material for: The Same, but Different: Shared Hallmarks and Divergent Drivers in Adult, Pediatric and Canine Glioma
Source: Curr Oncol. 2026 Sep 30;33(10):592. doi: 10.3390/curroncol33100592 (PMC13703710; doi:10.3390/curroncol33100592)
Supplement: Supplementary file 1 [file curroncol-33-00592-s001.zip › FigS2.pdf]

		cov	pid	81 . 1 . . . : . 160
1	IDH_human_WT	100.0%	100.0%	KRVEEFKLKQMWKSPNGTIRNILGGTVFREAIICKNIPRLVSGWVKPIIIGRHAYGDQYRATDFVVPGPGKVEITYTPSD
2	IDH_dog_WT	100.0%	96.9%	KRVEEFKLKQMWKSPNGTIRNILGGTVFREAIICKNIPRLVSGWVKPIIIGRHAYGDQYRATDFVVPGPGKVEITYTPSD
3	IDH_human_Mut	100.0%	99.8%	KRVEEFKLKQMWKSPNGTIRNILGGTVFREAIICKNIPRLVSGWVKPIIIGHHAYGDQYRATDFVVPGPGKVEITYTPSD
4	IDH_dog_Mut	100.0%	96.6%	KRVEEFKLKQMWKSPNGTIRNILGGTVFREAIICKNIPRLVSGWVKPIIIGCHAYGDQYRATDFVVPGPGKVEITYTPSD
	consensus/100%			KRVEEFKLKQMWKSPNGTIRNILGGTVFREAIICKNIPRLVSGWVKPIIIGpHAYGDQYRATDFVVPGPGKVEITYTPSD
	consensus/90%			KRVEEFKLKQMWKSPNGTIRNILGGTVFREAIICKNIPRLVSGWVKPIIIGpHAYGDQYRATDFVVPGPGKVEITYTPSD
	consensus/80%			KRVEEFKLKQMWKSPNGTIRNILGGTVFREAIICKNIPRLVSGWVKPIIIGpHAYGDQYRATDFVVPGPGKVEITYTPSD
	consensus/70%			KRVEEFKLKQMWKSPNGTIRNILGGTVFREAIICKNIPRLVSGWVKPIIIG+HAYGDQYRATDFVVPGPGKVEITYTPSD
