# Supplementary material for: The Same, but Different: Shared Hallmarks and Divergent Drivers in Adult, Pediatric and Canine Glioma
Source: Curr Oncol. 2026 Sep 30;33(10):592. doi: 10.3390/curroncol33100592 (PMC13703710; doi:10.3390/curroncol33100592)
Supplement: Supplementary file 1 [file curroncol-33-00592-s001.zip › FigS3.pdf]

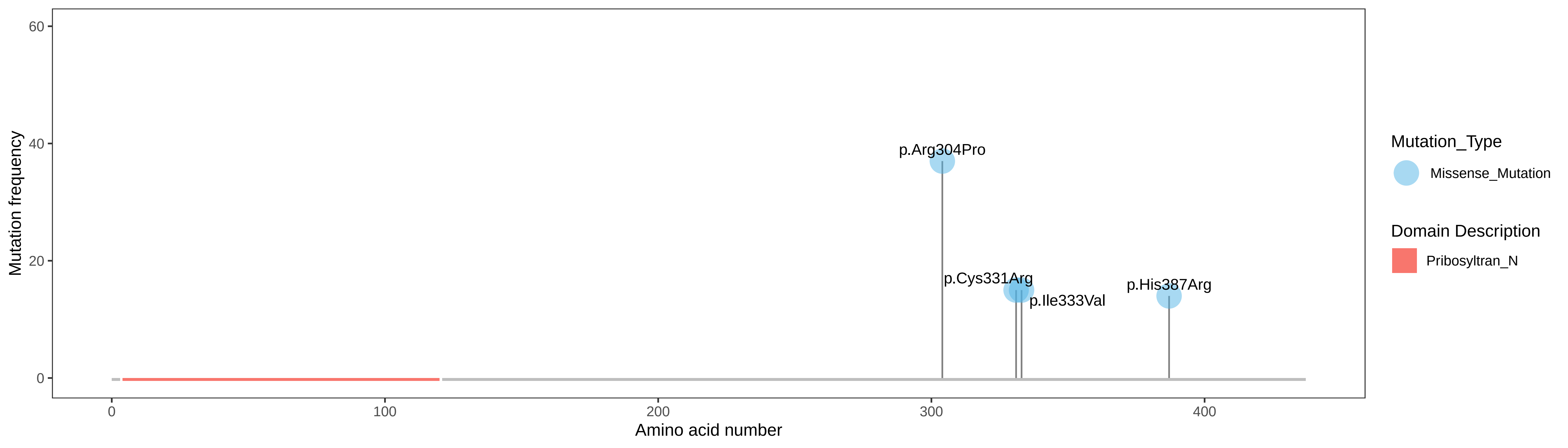

p.Arg304Pro
p.Cys331Arg
p.Ile333Val
p.His387Arg
Mutation frequency
60
40
20
0
0
100
200
300
400
Amino acid number
Mutation_Type
Missense_Mutation
Domain Description
Pribosyltran_N
