# Supplementary material for: The Same, but Different: Shared Hallmarks and Divergent Drivers in Adult, Pediatric and Canine Glioma
Source: Curr Oncol. 2026 Sep 30;33(10):592. doi: 10.3390/curroncol33100592 (PMC13703710; doi:10.3390/curroncol33100592)
Supplement: Supplementary file 1 [file curroncol-33-00592-s001.zip › FigS5_new.pdf]

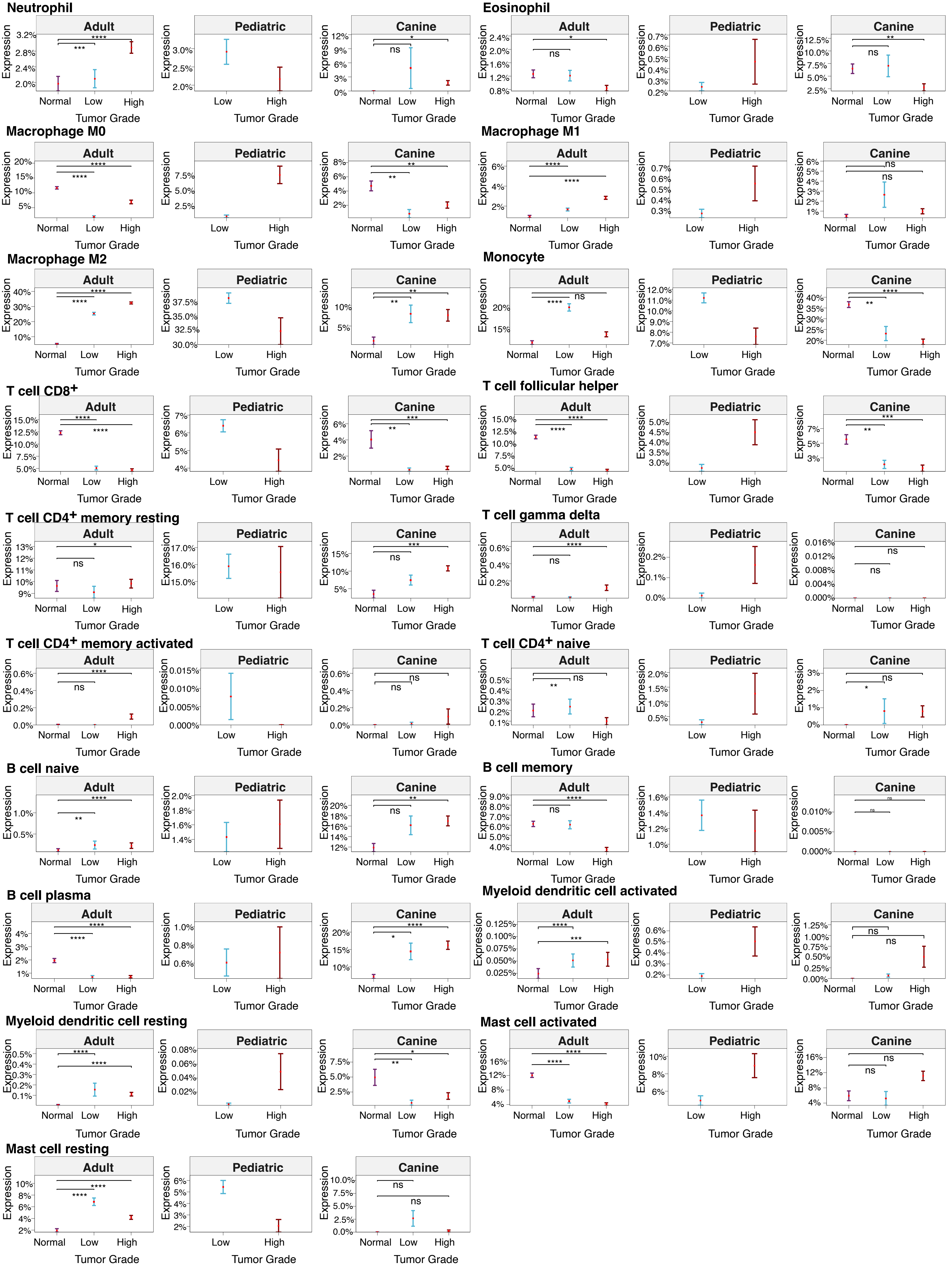
Neutrophil
Eosinophil
Macrophage M0
Macrophage M1
Macrophage M2
Monocyte
T cell CD8+
T cell follicular helper
T cell CD4+ memory resting
T cell gamma delta
T cell CD4+ memory activated
T cell CD4+ naive
B cell naive
B cell memory
B cell plasma
Myeloid dendritic cell activated
Myeloid dendritic cell resting
Mast cell activated
Mast cell resting
Adult
Pediatric
Canine
Expression
Normal
Low
High
Tumor Grade
ns
*
**
***
****
