# Supplementary material for: The Same, but Different: Shared Hallmarks and Divergent Drivers in Adult, Pediatric and Canine Glioma
Source: Curr Oncol. 2026 Sep 30;33(10):592. doi: 10.3390/curroncol33100592 (PMC13703710; doi:10.3390/curroncol33100592)

T cell regulatory (Tregs)

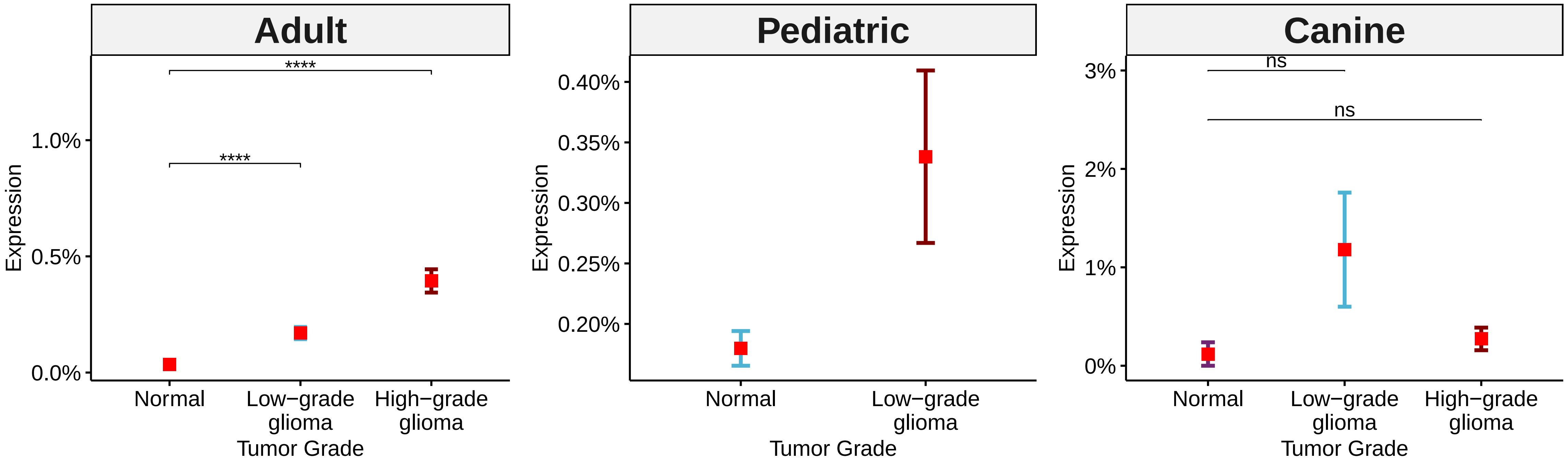

Supplement: Supplementary file 1 [file curroncol-33-00592-s001.zip › FigS6_new.pdf]
