# Supplementary material for: The Same, but Different: Shared Hallmarks and Divergent Drivers in Adult, Pediatric and Canine Glioma
Source: Curr Oncol. 2026 Sep 30;33(10):592. doi: 10.3390/curroncol33100592 (PMC13703710; doi:10.3390/curroncol33100592)
Supplement: Supplementary file 1 [file curroncol-33-00592-s001.zip › FigS7_new.pdf]

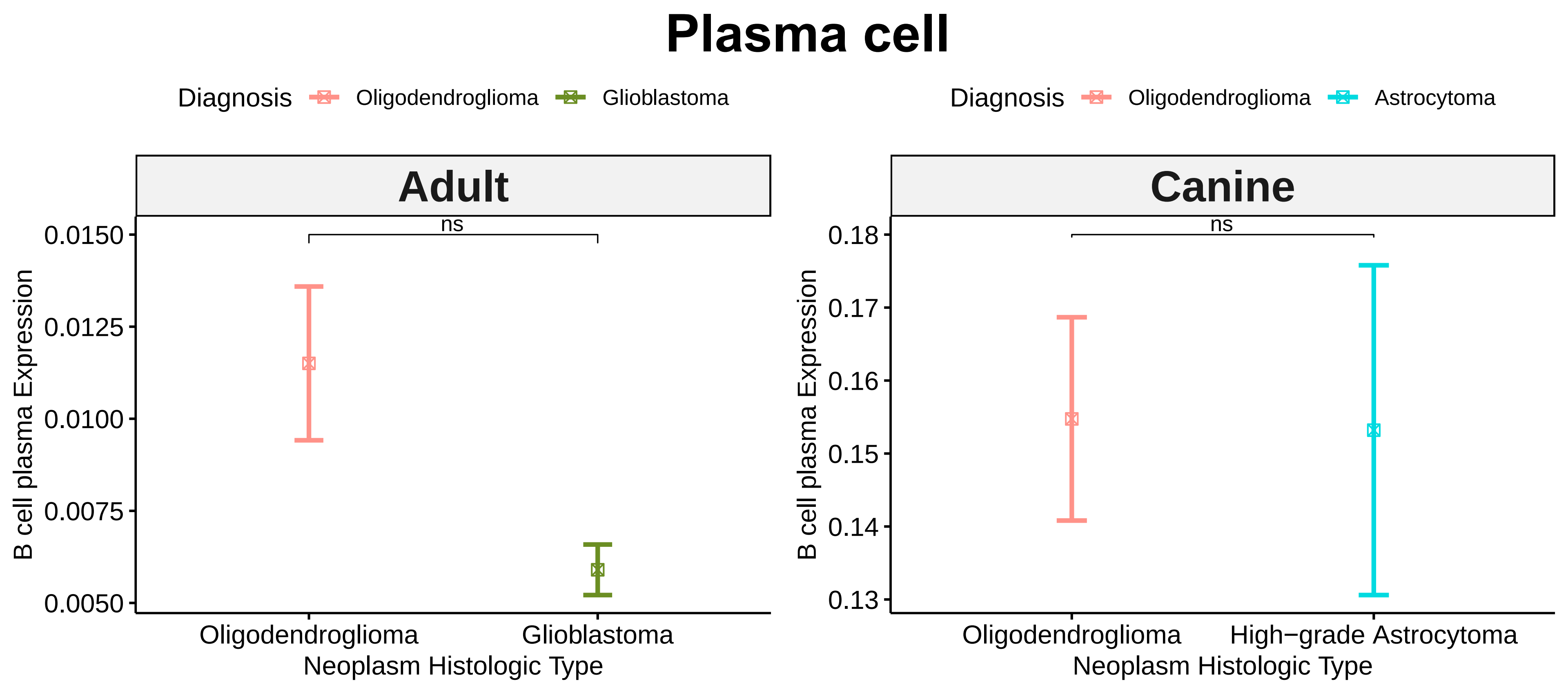

Plasma cell
Diagnosis
Oligodendroglioma
Glioblastoma
Adult
ns
0.0150
0.0125
0.0100
0.0075
0.0050
B cell plasma Expression
Oligodendroglioma
Glioblastoma
Neoplasm Histologic Type
Diagnosis
Oligodendroglioma
Astrocytoma
Canine
ns
0.18
0.17
0.16
0.15
0.14
0.13
B cell plasma Expression
Oligodendroglioma
High−grade Astrocytoma
Neoplasm Histologic Type
