# Supplementary material for: The Same, but Different: Shared Hallmarks and Divergent Drivers in Adult, Pediatric and Canine Glioma
Source: Curr Oncol. 2026 Sep 30;33(10):592. doi: 10.3390/curroncol33100592 (PMC13703710; doi:10.3390/curroncol33100592)
Supplement: Supplementary file 1 [file curroncol-33-00592-s001.zip › SUBMISSION_SUPPLEMENTAL ONLY.pdf]

SUPPLEMENTAL DATA

Table S1. Sample Summary – A summary of number of samples included for each analysis is provided.

Title	Adult		Pediatric		Canine	
	Glioma	Normal	Glioma	Normal	Glioma	Normal
**Expression:**						
RNAseq	606	464^	246	NA	39	12^
Cibersort & quanTIseq	606	464^	246	NA	39	12^
**Variations:**						
SNP calling	880	880*	118	118*	79	79*
CNV	891	891*	110	29**	79	22**
**Epigenomic:**						
Methylation	587***	NA	NA	NA	45	3

^ True-normal sample from healthy subject.
* adjacent-normal sample from matching patient.
** pooled reference created with provided number of adjacent-normal samples.
*** Methylation array data (only samples with assigned high/low grades were used).

Table S2. Canine Samples Metadata Table.

Table S3. Adult glioma (TCGA) Samples Metadata Table.

Table S4. Pediatric (KidsFirst) Samples Metadata Table.

Table S5. Adult normal (GTEx) Samples Metadata Table.

Table S6. Pathway gene details of Figure 1A.
See Excel Table.

Table S7. Pathway gene details of Figure 1B.
See Excel Table.

Table S8. IDH1 mutation status of glioma cases.

(By genomics data)	Low-Grade Glioma		High-Grade Glioma	
	IDH1	IDH1 or IDH2	IDH1	IDH1 or IDH2
**Adult**	181 / 213	192 / 213	187 / 745	197 / 745
**Pediatric**	1 / 92	1 / 92	1 / 24	2 / 24
**Canine**	2 /15	2 / 15	1 / 64	1 / 64

In dogs, there were 3 IDH1 mutations, no IDH2 mutations were present. These occurred in one brachycephalic dog (Boxer), one mixed-breed, and one purebred non-brachycephalic (Labrador retriever), and in one oligodendroglioma, one undefined glioma (“oligoastrocytoma”) and one astrocytoma. They were concentrated in low-grade glioma (2/15, 13.3%) and rather than high-grade glioma (1/64, 1.6%). All 3 canine IDH1mutations were R132C.

Table S9. Differential germline mutations: brachycephalic and non-brachycephalic dogs

Pathway	Brachycephalic (n = 50)	Non-Brachycephalic (n = 10)	Pvalue	OR	95% CI (upper)	95% CI (lower)	Adjusted Pvalue
TOR signaling	50	8	1.88E-02	15.84	903.1	1.135	0.41
NOTCH signaling	27	8	1.71E-01	0.2989	1.711	0.02821	0.41
Splicing	49	9	3.08E-01	5.217	434.1	0.06247	0.41
Histone modification	45	8	3.30E-01	2.213	16.82	0.1812	0.41
Cell cycle	50	10	3.42E-01	4.482	369.6	0.05418	0.41
Chromatin SWI/SNF complex	50	10	3.42E-01	4.482	369.6	0.05418	0.41
Chromatin histone modifiers	50	10	3.42E-01	4.482	369.6	0.05418	0.41
Chromatin other	50	10	3.42E-01	4.482	369.6	0.05418	0.41
Genome integrity	50	10	3.42E-01	4.482	369.6	0.05418	0.41
MAPK signaling	50	10	3.42E-01	4.482	369.6	0.05418	0.41
NFKB signaling	50	10	3.42E-01	4.482	369.6	0.05418	0.41
Other	50	10	3.42E-01	4.482	369.6	0.05418	0.41
Other signaling	50	10	3.42E-01	4.482	369.6	0.05418	0.41
PI3K signaling	50	10	3.42E-01	4.482	369.6	0.05418	0.41
Protein homeostasis/ubiquitination	50	10	3.42E-01	4.482	369.6	0.05418	0.41
RNA abundance	50	10	3.42E-01	4.482	369.6	0.05418	0.41
RTK signaling	50	10	3.42E-01	4.482	369.6	0.05418	0.41
TGFB signaling	50	10	3.42E-01	4.482	369.6	0.05418	0.41
Transcription factor	50	10	3.42E-01	4.482	369.6	0.05418	0.41
Wnt/B-catenin signaling	50	10	3.42E-01	4.482	369.6	0.05418	0.41
Epigenetics DNA modifiers	34	6	7.18E-01	1.408	6.946	0.2552	0.82
Apoptosis	43	9	1.00E+00	0.6865	6.563	0.01365	1
Immune signaling	45	9	1.00E+00	1	10.67	0.01905	1
Metabolism	13	3	1.00E+00	0.8226	5.655	0.1566	1

SUPPLEMENTAL FIGURES

Figure S1. Copy number variation in genes of the 3 GBM canonical pathways (PI3K/MAPK, Rb1 and p53) in canine glioma (low- and high-grade).

Figure S2. Multiple sequence alignment of wild-type and mutant IDH sequences (human and canine), highlighting position 132.

Figure S3. 87 non-synonymous germline mutations in PRPS2, identified in 37 brachycephalic dogs.

Figure S4. Germline mutational burden within oncogenic signaling pathways in brachycephalic (red) and non-brachycephalic (blue) dogs with glioma.

Figure S5. Deconvolution of immune signatures computed by Cibersort [82].

Figure S6. Deconvolution of Treg signatures computed by quanTIseq [83].

Figure S7. Plasma cell infiltration in adult oligodendroglioma versus glioblastoma, and in canine oligodendroglioma versus high-grade astrocytoma.
